# Mitofusin-2 suppresses tumor immune escape through EGFR/STAT3-mediated PD-L1 transcription

**DOI:** 10.1038/s41419-026-08668-3

**Published:** 2026-03-27

**Authors:** Yan Liu, Ningning Wang, Zhenhua Li, Na Li, Fu Hui, Xinlei Wang, Gaoyan Tang, Qingyun Zhang, Guohua Yu, Shuzhen Liu, Yanhong Ding

**Affiliations:** 1 Oncology Laboratory of the First Affiliated Hospital, Weifang People’s Hospital, Shandong Second Medical University, Weifang, 261000 China; 2 Shanghai Clinical Research and Trial Center, ShanghaiTech University, Shanghai, 200120 China; 3 Clinical College of Shandong Second Medical University, Weifang, 261000 China

**Keywords:** Cancer microenvironment, Immunosuppression

## Abstract

Immune evasion driven by aberrant PD-L1 expression poses a significant challenge to the efficacy of cancer immunotherapy. Although Mitofusin-2 (MFN2) is recognized for its role in tumor suppression, its specific contribution to the regulation of immune escape remains poorly understood. Here, we integrated analyses of public datasets, clinical specimens, and mechanistic experiments in multiple cancer cell lines, immunocompetent mouse models, and patient-derived organoids. A combination of molecular assays and single-cell transcriptomic reanalysis was employed to elucidate how MFN2 influences tumor immune escape. MFN2 expression was markedly reduced in various cancers and inversely correlated with PD-L1 levels and immunosuppressive gene signatures. Functional assays demonstrated that MFN2 suppresses PD-L1 transcription by limiting EGFR-dependent activation and nuclear translocation of STAT3. Loss of MFN2 enhanced PD-L1 expression, impaired CD8^+^ T-cell cytotoxicity, and accelerated tumor growth in immunocompetent mice. Conversely, restoration of MFN2 or pharmacological inhibition of STAT3 decreased PD-L1 expression and reactivated antitumor immunity. Our findings identify MFN2 as a critical suppressor of tumor immune evasion through the EGFR/STAT3–PD-L1 signaling pathway. Targeting this axis may offer a novel strategy to enhance the efficacy of PD-1/PD-L1–based immunotherapy.

## Introduction

Tumor cells employ multifaceted mechanisms to circumvent immune surveillance, a dynamic process collectively termed cancer immunoediting [1]. This process frequently involves the modulation of immune checkpoint molecules, most notably programmed cell death ligand-1 (PD-L1) [2, 3]. The interaction between programmed cell death-1 (PD-1), typically expressed on activated T cells, and PD-L1 on the surface of tumor cells suppresses T-cell activation and impairs cytotoxicity, thereby facilitating immune escape [4, 5]. Aberrant or sustained PD-L1 expression is a hallmark of multiple malignancies—including lung cancer, melanoma, glioma, and breast cancer—where it fosters the establishment of an immunosuppressive tumor microenvironment (TME) [6].

While therapeutic antibodies targeting the PD-1/PD-L1 axis, such as nivolumab and atezolizumab, have revolutionized the clinical management of non-small cell lung cancer, renal cell carcinoma, and various other solid tumors [7–9], the frequent emergence of acquired resistance remains a formidable challenge [10, 11]. Such resistance is often characterized by compensatory PD-L1 upregulation in tumor cells, which further blunts antitumor immunity. Therefore, elucidating the precise molecular mechanisms governing PD-L1 expression is imperative for overcoming immune evasion and optimizing the clinical efficacy of current immunotherapies.

Mitochondria are highly dynamic organelles that undergo continuous fission and fusion to maintain cellular homeostasis and metabolic adaptation [12]. Mitofusin-2 (MFN2), a GTPase anchored to the outer mitochondrial membrane (OMM), serves as a pivotal mediator of mitochondrial fusion [13]. Dysfunctional MFN2 has been implicated in a broad spectrum of pathological conditions, including metabolic disorders, neuropathies, and various malignancies [14–17]. Beyond its canonical roles in mitochondrial dynamics, recent evidence suggests that MFN2 interacts with the epidermal growth factor receptor (EGFR) to modulate EGFR/STAT3 signaling [17–19] —a pathway well-characterized for promoting tumor progression and immune evasion via the transcriptional activation of PD-L1 [18].

Despite these insights, the specific role of MFN2 in regulating tumor immune escape remains poorly defined. In particular, whether MFN2 directly governs PD-L1 expression and shapes the tumor immune microenvironment (TIME) through the EGFR/STAT3 axis has not been elucidated.

Our research group has a longstanding interest in investigating the functions of MFN2 [20, 21]. In the present study, we investigated the regulatory functions of MFN2 in tumor immune evasion. By integrating analyses of public datasets, clinical specimens, and mechanistic models, we examined the impact of MFN2 on PD-L1 expression and antitumor immunity. Using lung adenocarcinoma (LUAD) and kidney renal clear cell carcinoma (KIRC) as representative models, we combined multi-omic profiling, in vitro and in vivo experiments, single-cell transcriptomic reanalysis, and patient-derived organoids to delineate the MFN2–EGFR/STAT3–PD-L1 axis. Our findings uncovered a previously unrecognized link between mitochondrial dynamics and immune checkpoint regulation, providing mechanistic insights and a potential therapeutic strategy to overcome immune escape and resistance to PD-1/PD-L1 blockade.

## Results

### Inverse correlation between MFN2 and PD-L1 expression is associated with poor overall survival in lung and kidney cancer patients

**Fig. 1:**
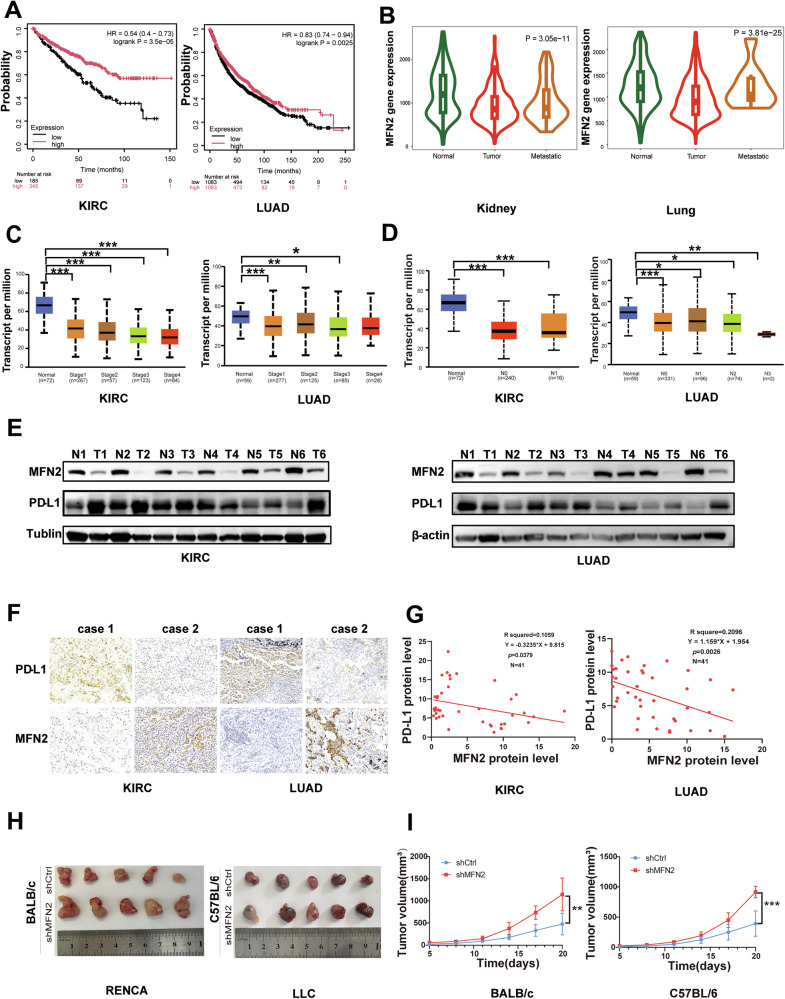
MFN2 and PD-L1 expression are inversely correlated, with lower MFN2 linked to poorer overall survival in lung and kidney cancer patients. **A** Kaplan–Meier survival analysis showing overall survival in lung and kidney cancer patients with high and low MFN2 expression. Kaplan-Meier analysis along with log-rank test. **B** TNM plot analysis showing MFN2 mRNA expression level in primary and metastatic kidney and lung tumors, TNM plot analysis along with Dunn test. TCGA analysis showing the association between MFN2 expression and clinical stage (**C**) and lymph node involvement (**D**) based on UALCAN database. **E** Representative images showing MFN2 protein levels in six pairs of human KIRC and LUAD tumor tissues and adjacent normal tissues. Tubulin was used as a loading control. **F** Representative IHC staining of MFN2 and PD-L1 in KIRC and LUAD tumor tissues from selected MFN2-High and MFN2-Low patients. Scale bar: 100 µm. **G** Correlation between MFN2 and PD-L1 positivity rates in KIRC and LUAD tumor tissue samples (n = 41), Pearson’s correlation analysis was used to calculate the R and p values. **H**,**I** Analysis of tumor growth in xenograft mouse models using RENCA cells in BALB/C mice (n = 5 per group) and LLC cells in C57BL/6 mice (n = 5 per group) by one-way ANOVA. Data are presented as mean ± SEM. *P < 0.05, **P < 0.01, ***P < 0.001.

MFN2 is frequently downregulated across multiple tumor types and is associated with unfavorable patient prognosis [22]. Similarly, elevated PD-L1 expression has been linked to poor clinical outcomes in various malignancies, including lung, head and neck, kidney, and gastric carcinomas [23–25]. In this study, Kaplan–Meier survival analysis demonstrated that patients with LUAD or KIRC exhibiting low MFN2 expression had significantly shorter overall survival (OS) compared to those with high MFN2 expression (Fig. 1A). Analysis of gene expression data via the TNMplot platform (https://tnmplot.com/analysis/) further confirmed that MFN2 was significantly downregulated in primary kidney and lung tumor tissues, with even more pronounced suppression observed in corresponding metastatic lesions (Fig. 1B). Furthermore, analysis of The Cancer Genome Atlas (TCGA) datasets revealed that MFN2 expression levels were significantly correlated with clinical stage (Fig. 1C), lymph node involvement (Fig. 1D), and histological grade (Supplementary Fig. S1A).

To validate these bioinformatic findings, we examined the protein levels of MFN2 and PD-L1 in histologically confirmed tumor tissues and adjacent normal tissues using western blotting. MFN2 expression was markedly decreased in tumor tissues, whereas PD-L1 levels were significantly elevated in both KIRC and LUAD samples (Fig. 1E). Immunohistochemical (IHC) analysis of clinical specimens further revealed a robust inverse correlation between MFN2 and PD-L1 protein expression (Fig. 1F). Quantitative assessment using standardized scoring systems indicated that high MFN2 expression was associated with reduced PD-L1 positivity, whereas low MFN2 expression correlated with elevated PD-L1 immunoreactivity (Fig. 1G).

Having established the clinical correlation, we next investigated the functional role of MFN2 in vivo using subcutaneous tumor xenograft models in immunocompetent BALB/c and C57BL/6 mice. Successful knockdown of MFN2 in Renca and LLC murine cell lines led to a concomitant increase in PD-L1 expression (Fig. S1B). Notably, MFN2 deficiency significantly accelerated tumor growth in both the BALB/c and C57BL/6 models (Fig. 1H,I). To further characterize the immune landscape, we evaluated additional immune-related markers, including MHC-I (HLA-A, HLA-B, HLA-C) and CD47. While MFN2 depletion had negligible effects on MHC-I levels, it markedly upregulated CD47 expression (Fig. S1C), suggesting that MFN2 loss reshapes the immune microenvironment through multidimensional mechanisms. Collectively, these findings suggest that MFN2 serves as a negative regulator of PD-L1-mediated immune evasion, highlighting its potential as both a predictive biomarker and a therapeutic target in KIRC and LUAD.

### MFN2 regulates PD-L1 expression across multiple cancer cell lines

**Fig. 2:**
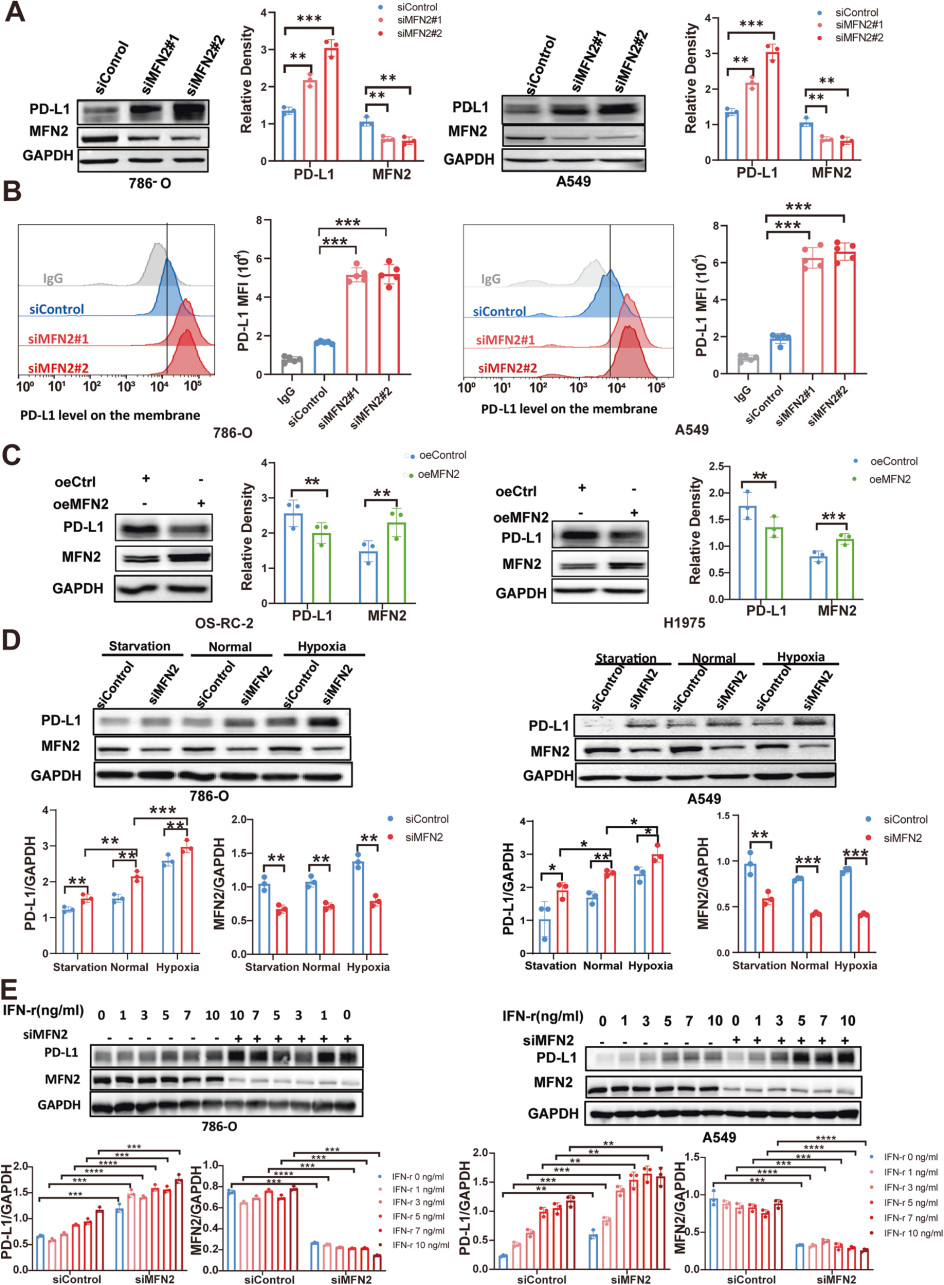
MFN2 regulates PD-L1 expression in multiple cancer cells. **A** Western blot analysis showing PD-L1 protein levels following MFN2 knockdown in 786-O and A549 cells by One-way ANOVA followed by Tukey’s test (n = 3). **B** Flow cytometric analysis of PD-L1 surface expression in MFN2-knockdown 786-O and A549 cells by One-way ANOVA followed by Tukey’s test (n = 5). **C** Western blot analysis of PD-L1 protein expression in OS-RC-2 and H1975 cells following MFN2 overexpression by Student’s *t* test (n = 3). **D** Western blot analysis showing PD-L1 protein levels following MFN2 knockdown in 786-O and A549 cells under starvation, hypoxia, and normal conditions by One-way ANOVA followed by Tukey’s test (n = 3). **E** Western blot analysis showing PD-L1 protein levels following MFN2 knockdown in 786-O and A549 cells under IFN-γ stimulation by One-way ANOVA followed by Tukey’s test (n = 3). Data were shown as mean ± SEM; *P < 0.05, **P < 0.01, ***P < 0.001, ****P < 0.0001.

To evaluate the role of MFN2 in modulating PD-L1 expression, we first transiently transfected MFN2-targeting siRNAs into KIRC (786-O) and LUAD (A549) cells. Western blot analysis confirmed that siRNA-mediated knockdown of MFN2 led to a significant increase in PD-L1 protein levels in both cell lines (Fig. 2A). This upregulation of PD-L1 following MFN2 silencing was consistently recapitulated in a broad panel of other malignancies, including various lung cancer cell lines (H1299, HCC827, PC-9, H1703, and H1975) (Fig. S2A), as well as kidney cancer (769-P) (Fig. S2B) and breast cancer (MDA-MB-231) models (Fig. S2C).

Since membrane-localized PD-L1 is the functional isoform that suppresses antitumor immunity by binding to the PD-1 receptor on T cells [2], we next examined whether MFN2 affects the surface distribution of PD-L1. Flow cytometry revealed that MFN2 knockdown significantly increased the surface expression of PD-L1 in both 786-O and A549 cells (Fig. 2B), with similar results observed in 769-P cells (Fig. S2D). To confirm the specificity of this regulatory effect, we performed rescue experiments. Western blot analysis showed that the reintroduction of MFN2 successfully restored PD-L1 expression to baseline levels in OS-RC-2 and H1975 cells (Fig. 2C). Consistently, flow cytometric analysis confirmed that MFN2 overexpression reduced surface PD-L1 levels in OS-RC-2 cells (Fig. S2E).

Tumor progression frequently induces a hostile tumor microenvironment (TME) characterized by hypoxia and nutrient deprivation [26]. To investigate whether the regulatory effect of MFN2 on PD-L1 persists under these stress conditions, we conducted western blot analyses in 786-O and A549 cells. Our results demonstrated that MFN2 deficiency markedly elevated PD-L1 levels even in hypoxic or nutrient-deprived environments. Notably, while nutrient deprivation alone appeared to attenuate PD-L1 expression, hypoxic exposure promoted its upregulation—observations that are fully consistent with previous reports [27, 28] (Fig. 2D). Furthermore, given that PD-L1 is a highly inducible protein, we examined the interplay between MFN2 and IFN-γ, a potent inducer of PD-L1 [29]. We found that MFN2 knockdown in A549 and 786-O cells further sensitized the cells to IFN-γ, leading to a synergistic upregulation of PD-L1 expression (Fig. 2E). Collectively, these data suggest that MFN2 serves as a robust negative regulator of both basal and induced PD-L1 expression across diverse oncogenic contexts.

### Loss of MFN2 Impairs CD8^+^ T-cell infiltration and cytotoxic activity

**Fig. 3:**
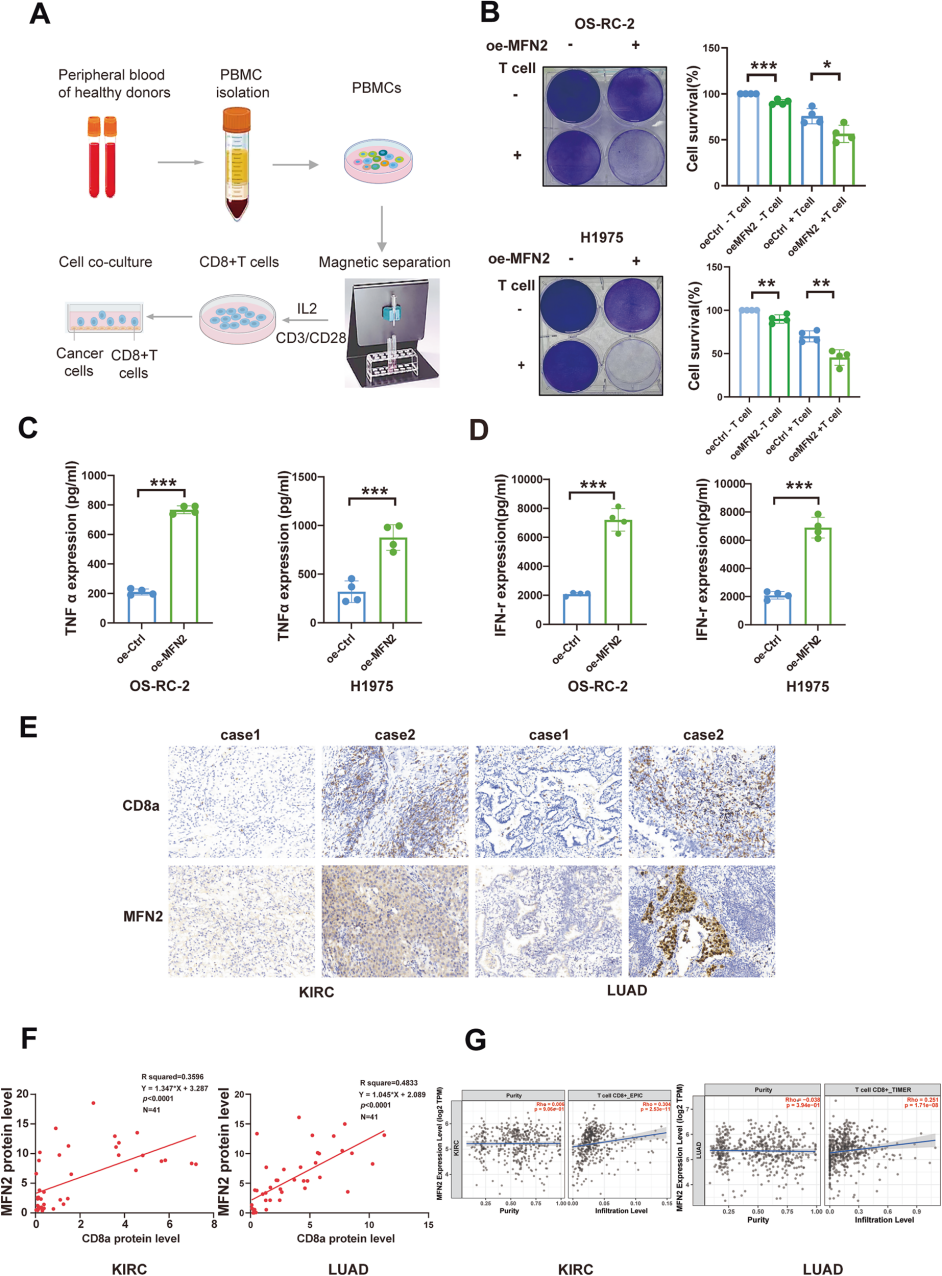
Loss of MFN2 reduces CD8^+^ T cell infiltration and activity. **A** Illustration of the in vitro procedure for isolating human CD8^+^ T cells and co-cultivating them with cancer cells. **B** Crystal violet staining of oe-control or oe-MFN2 OS-RC-2 and H1975 cells co-cultured with activated T cells for 48 h (n = 4). The ratio of OS-RC-2 and H1975 cells to T cells was 1:3. ELISA analysis of TNF-α (**C**) and IFN-γ (**D**) expression in the supernatant after co-culture of activated T cells with OS-RC-2 and H1975 oe-control or oe-MFN2 cells (n = 4). **E** Representative IHC staining of MFN2 and CD8 in KIRC and LUAD tumor tissues from selected MFN2-High and MFN2-Low patients. Scale bar: 100 µm. **F** Correlation between MFN2 and CD8a positivity rates in KIRC and LUAD tumor tissue samples (n = 41). Pearson’s correlation analysis was used to calculate the R and p values. **G** TIMER database analysis showing the correlation between MFN2 expression and CD8^+^ T cell infiltration in KIRC and LUAD. Data were shown as mean ± SEM; *P < 0.05, **P < 0.01, ***P < 0.001, by One-way ANOVA followed by Tukey’s test (B) or Student’s *t* test (**C**, **D**).

To determine whether the MFN2-mediated regulation of PD-L1 directly influences tumor immune evasion, we first performed T-cell killing assays by co-culturing tumor cells with activated human peripheral blood mononuclear cells (PBMCs) [29] (Fig. 3A). Notably, overexpression of MFN2 in OS-RC-2, H1975 (Fig. 3B), A549, and 786-O cells (Fig. S3A) significantly sensitized these cancer cells to T-cell-mediated killing. Consistently, PBMCs co-cultured with MFN2-overexpressing cancer cells exhibited significantly higher secretion levels of IFN-γ and TNF-α compared to the control groups (Fig. 3C, D) (Fig. S3B, C). These findings demonstrate that MFN2 limits immune evasion by downregulating PD-L1, thereby restoring T-cell effector functions.

To bridge the gap between in vitro observations and clinical reality, we performed immunohistochemical (IHC) analysis on patient-derived tumor specimens. This analysis revealed a robust positive correlation between MFN2 expression levels and the density of infiltrating CD8^+^ T cells in both LUAD and KIRC tissues (Fig. 3E, F). This clinical association was further corroborated by bioinformatic analysis using the TIMER database, which demonstrated a strong positive link between MFN2 levels and CD8^+^ T-cell infiltration across multiple cohorts, including LUAD, KIRC (Fig. 3G), and various BRCA subtypes (Fig. S3D–G).

Finally, we explored the translational potential of our findings by analyzing clinical datasets of patients receiving anti-PD-L1 immunotherapy. Leveraging the KM-plotter database, we found that high MFN2 expression was significantly associated with superior therapeutic outcomes, as evidenced by improved progression-free survival (PFS) and overall survival (OS) (Fig. S3H, I). Collectively, these results demonstrate that MFN2 is a critical determinant of the tumor immune microenvironment (TIME), playing a pivotal role in the recruitment, maintenance, and functional activation of CD8^+^ T cells.

**Fig. 4:**
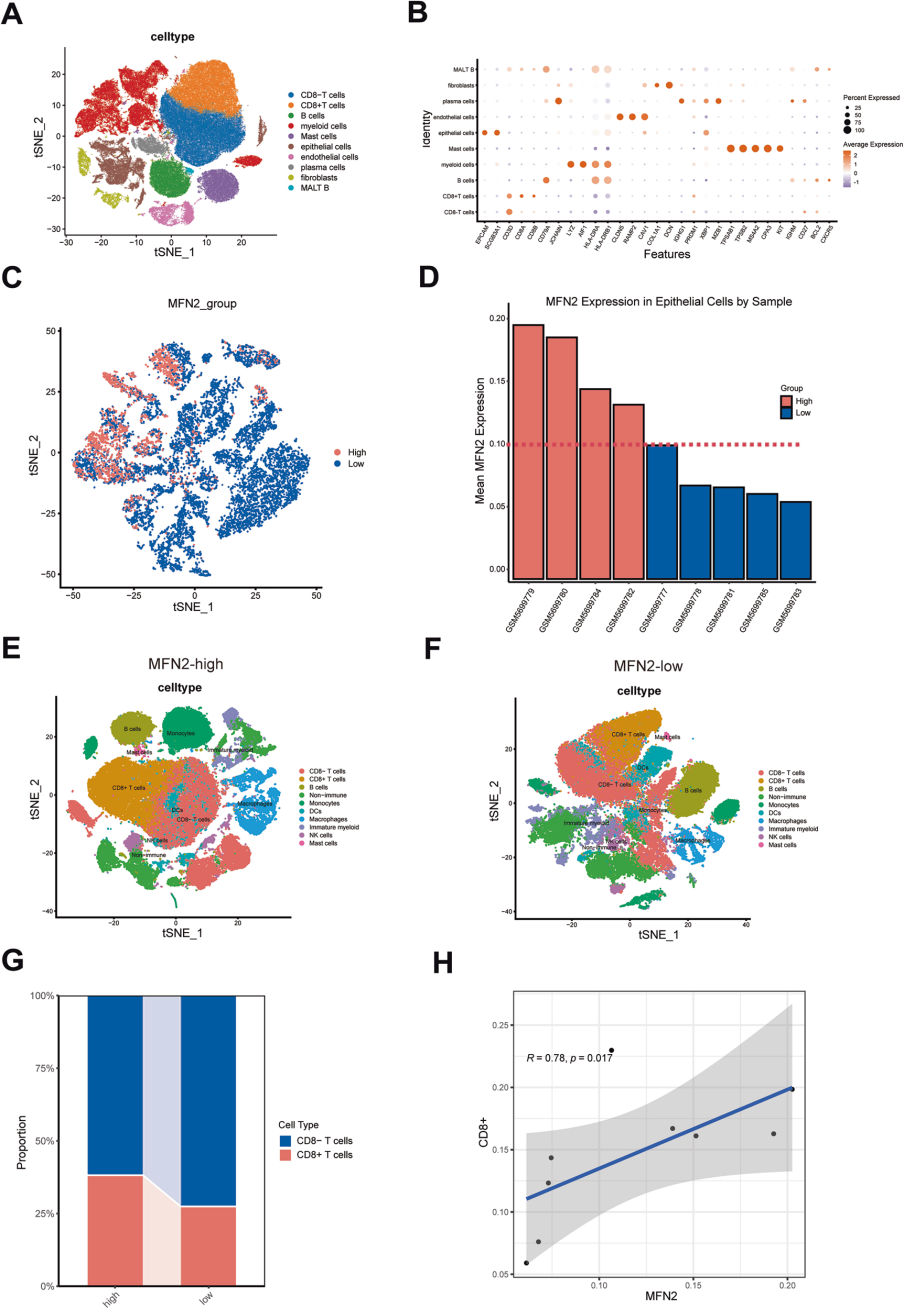
MFN2 enhances intratumoral infiltration of CD8^+^ T cells. **A** Clustered cells colored by cell type. **B** Dot plot of lowest adjusted p-value cell type-specific markers used to assign cell identity. **C**, **D** MFN2 Expression in Epithelial Cells of Samples. **E**, **F** Immune landscape of MFN2-high and MFN2-low samples respectively. **G** MFN2 enhances intratumoral infiltration of CD8^+^ T cells. **H** MFN2 was associated with infiltration of CD8^+^ T cells.

To further validate the impact of MFN2 on CD8^+^ T-cell infiltration in tumors, we reanalyzed a single-cell dataset comprising eight lung cancer samples obtained from the GEO database. After rigorous quality control and batch-effect correction, we performed dimensionality reduction, clustering, and cell-type annotation to characterize the tumor immune microenvironment. Ten distinct cell types were identified, including epithelial cells, endothelial cells, myeloid cells, fibroblasts, MALTB cells, plasma cells, mast cells, B cells, as well as CD8⁻ and CD8^+^ T cells (Fig. 4A). The expression of key marker genes for each cell type was visualized using bubble plots (Fig. 4B).

To explore the influence of MFN2 on the TIME, we stratified the eight samples into “MFN2-high” and “MFN2-low” groups based on the median expression of MFN2 within the epithelial (tumor) cell population (Fig. 4C, D). Immune cells from each group were subsequently re-clustered for downstream analysis (Fig. 4E, F). This stratification revealed a significant enrichment of CD8^+^ T cells in the MFN2-high group compared to the MFN2-low group. Moreover, MFN2 expression levels in tumor cells exhibited a significant positive correlation with the abundance of intratumoral CD8^+^ T cells (Fig. 4G, H). Taken together, our multi-scale analysis demonstrates that MFN2 reshapes the tumor immune landscape and serves as a critical driver for the infiltration and activation of CD8^+^ T cells.

### MFN2 deficiency activates the EGFR/STAT3 axis to drive PD-L1 transcription

**Fig. 5:**
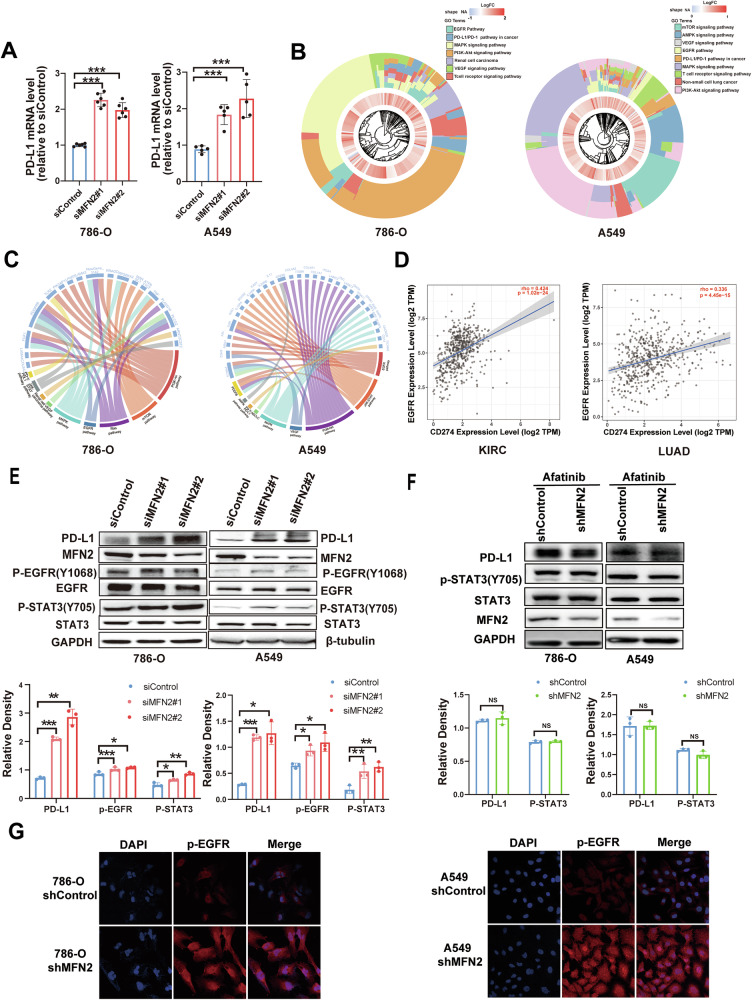
Loss of MFN2 activates the EGFR/STAT3 pathway to upregulate PD-L1. **A** RT-PCR analysis showing PD-L1 mRNA expression levels following MFN2 silencing in 786-O and A549 cells by One-way ANOVA followed by Tukey’s test. **B** Chord diagram depicting differentially expressed genes between MFN2 high and low expression groups in TCGA-KIRC and TCGA-LUAD cohorts, along with their association to corresponding oncogenic pathways. **C** Pathway analysis showing altered signaling pathways upon MFN2 knockdown in 786-O and A549 cells. **D** TIMER database analysis showing the correlation between PD-L1 and EGFR expression in KIRC and LUAD. **E** Western blot analysis of EGFR pathway proteins in MFN2-knockdown 786-O and A549 cells using the indicated antibodies by One-way ANOVA followed by Tukey’s test (n = 3). **F** Western blot analysis of indicated proteins in MFN2 knockdown 786-O and A549 cells treated with the EGFR inhibitor Afatinib by One-way ANOVA followed by Tukey’s test (n = 3). **G** Immunofluorescence detection of p-EGFR expression following MFN2 knockdown in 786-O and A549 cells. Data were shown as mean ± SEM; *P < 0.05, **P < 0.01, ***P < 0.001. NS no significance.

To further investigate the regulatory impact of MFN2 on PD-L1 expression, we first measured *CD274* (PD-L1) mRNA levels following MFN2 knockdown. RT-qPCR analysis demonstrated that MFN2 silencing significantly elevated PD-L1 transcript levels in 786-O and A549 cells (Fig. 5A), with consistent increases observed in H1299, H1703, and PC-9 cells (Fig. S4A). These results suggest that MFN2-mediated regulation of PD-L1 occurs at the transcriptional level.

To delineate the precise molecular mechanisms by which MFN2 governs PD-L1 transcription, we performed WikiPathways enrichment analysis using transcriptomic profiles from the TCGA-LUAD and TCGA-KIRC cohorts, stratified by MFN2 expression levels (Fig. 5B). The analysis revealed that MFN2 expression was closely associated with several key oncogenic signaling pathways, including VEGF, EGFR, PD-L1/PD-1 immune checkpoint, MAPK, PI3K-AKT-mTOR, and T-cell receptor signaling. To validate these bioinformatic predictions, we performed RNA sequencing (RNA-seq) on MFN2-silenced A549 and 786-O cells. Transcriptomic profiling identified significant gene expression shifts following MFN2 knockdown, with functional enrichment analysis highlighting the upregulation of the EGFR signaling pathway and its downstream STAT signaling components (Fig. 5C), thereby corroborating the TCGA findings. Notably, STAT3 signaling was significantly enriched in MFN2-deficient cells. Furthermore, analysis of the public TIMER database revealed significant positive correlations between *CD274* (PD-L1) and *EGFR* expression in both LUAD and KIRC (Fig. 5D).

These transcriptomic findings were further substantiated by western blot analysis. In A549 and 786-O cells (Fig. 5E), MFN2 knockdown led to elevated phosphorylation levels of EGFR (p-EGFR) and its downstream effector STAT3 (p-STAT3). To determine the specificity of this regulatory axis, we employed a panel of kinase inhibitors. Treatment with Afatinib (a second-generation irreversible EGFR inhibitor) (Fig. 5F) or Gefitinib (a selective EGFR tyrosine kinase inhibitor) (Fig. S4B) effectively abolished the MFN2-deficiency-induced p-STAT3 activation and subsequent PD-L1 upregulation. Notably, multi-kinase inhibitors such as Lenvatinib (targeting VEGFR, FGFR, and PDGFR) or Cabozantinib (targeting VEGFR and MET) failed to suppress the elevated p-STAT3 levels induced by MFN2 knockdown (Fig. S4C). This pharmacological contrast underscores the pivotal and specific role of EGFR in mediating the downstream immune-suppressive program. Moreover, immunofluorescence staining confirmed that MFN2 depletion increased p-EGFR expression levels (Fig. 5G).

Furthermore, to explore the nature of this regulation, we performed co-immunoprecipitation (Co-IP) assays. The results indicated no direct physical interaction between MFN2 and p-EGFR (Fig. S4D), suggesting that MFN2 restricts EGFR activation through an indirect mechanism, likely involving mitochondrial-to-nuclear retrograde signaling or altered organelle crosstalk [15]. Collectively, these findings confirm that MFN2 depletion promotes PD-L1 expression and a broader immunosuppressive phenotype primarily through the EGFR/STAT3 signaling axis.

### MFN2 regulates PD-L1 transcription by facilitating the nuclear accumulation of STAT3

**Fig. 6:**
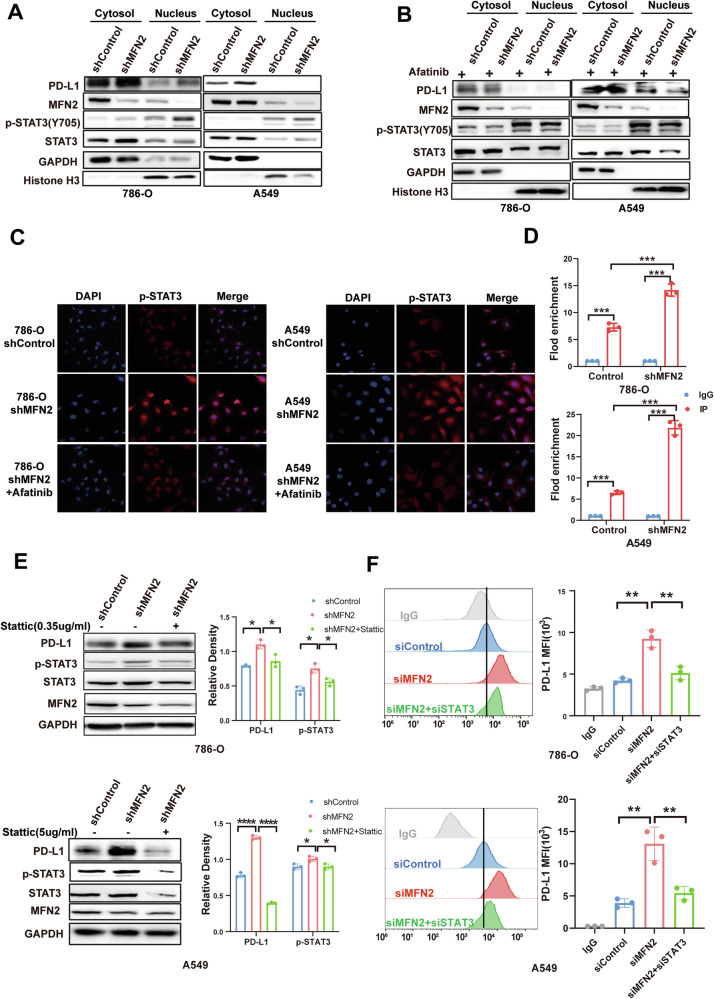
MFN2 regulated PD-L1 through facilitating STAT3 nuclear accumulation. **A** Western blot analysis showing p-STAT3, STAT3, and PD-L1 distribution in nuclear and cytoplasmic fractions of 786-O and A549 cells following MFN2 knockdown. **B** Western blot analysis of nuclear and cytoplasmic fractions from 786-O and A549 cells treated with the EGFR inhibitor afatinib, probed for p-STAT3, STAT3, and PD-L1. **C** Immunofluorescence analysis of p-STAT3 in 786-O and A549 cells after MFN2 knockdown, demonstrating reversal of p-STAT3 nuclear translocation by afatinib. **D** CHIP analysis of STAT3 binding to PD-L1 in 786-O and A549 cells following MFN2 knockdown, One-way ANOVA followed by Tukey’s test. **E** Western blot analysis of PD-L1 expression following Stattic treatment in MFN2-knockdown 786-O and A549 cells, One-way ANOVA followed by Tukey’s test (n = 3). **F** Flow cytometric analysis of PD-L1 surface expression in MFN2-knockdown 786-O and A549 cells following stattic treatment, One-way ANOVA followed by Tukey’s test (n = 3). Data were shown as mean ± SEM; *P < 0.05, **P < 0.01, ***P < 0.001, ****P < 0.0001.

To further elucidate how MFN2 deficiency drives the transcriptional upregulation of PD-L1, we investigated the subcellular localization of key signaling mediators. Western blot analysis of nuclear and cytoplasmic fractions demonstrated that MFN2 silencing promoted the nuclear translocation of phosphorylated STAT3 (p-STAT3) in 786-O and A549 cells (Fig. 6A). To determine whether this translocation was dependent on the activated EGFR signaling described above, we employed pharmacological inhibition. Notably, the nuclear accumulation of p-STAT3 induced by MFN2 knockdown was effectively abrogated upon treatment with the EGFR inhibitor afatinib (Fig. 6B).

These findings were further substantiated by confocal immunofluorescence microscopy (Fig. 6C). In untreated A549 and 786-O cells, p-STAT3 fluorescence was weak and largely cytoplasmic. Upon MFN2 knockdown, p-STAT3 fluorescence intensity increased markedly and was predominantly localized in the nucleus. Treatment with afatinib reversed the enhanced nuclear localization of p-STAT3 induced by MFN2 depletion. To determine whether the nuclear-localized p-STAT3 directly modulates CD274 (PD-L1) at the transcriptional level, we performed Chromatin Immunoprecipitation (ChIP) assays. The results demonstrated that MFN2 depletion significantly enriched the binding of p-STAT3 to the PD-L1 promoter region in both A549 and 786-O cells (Fig. 6D). To evaluate the functional necessity of STAT3 signaling in MFN2-mediated PD-L1 regulation, we employed multiple inhibitory strategies. We utilized Stattic to block STAT3 phosphorylation (Fig. 6E) and S3I-201, a selective inhibitor that prevents STAT3 DNA-binding activity (Fig. S4E). Western blot analysis revealed that pharmacological inhibition of STAT3 by either agent effectively abolished the upregulation of PD-L1 triggered by MFN2 knockdown. Finally, flow cytometric analysis of membrane-bound PD-L1 (Fig. 6F) confirmed that the surface expression of this checkpoint was neutralized upon STAT3 inhibition. Collectively, these results establish that MFN2 regulates PD-L1 expression through an EGFR-dependent p-STAT3 nuclear translocation and subsequent transcriptional activation program.

### Pharmacological inhibition of STAT3 suppresses MFN2 deficiency-induced tumor growth and immune evasion in vivo

**Fig. 7:**
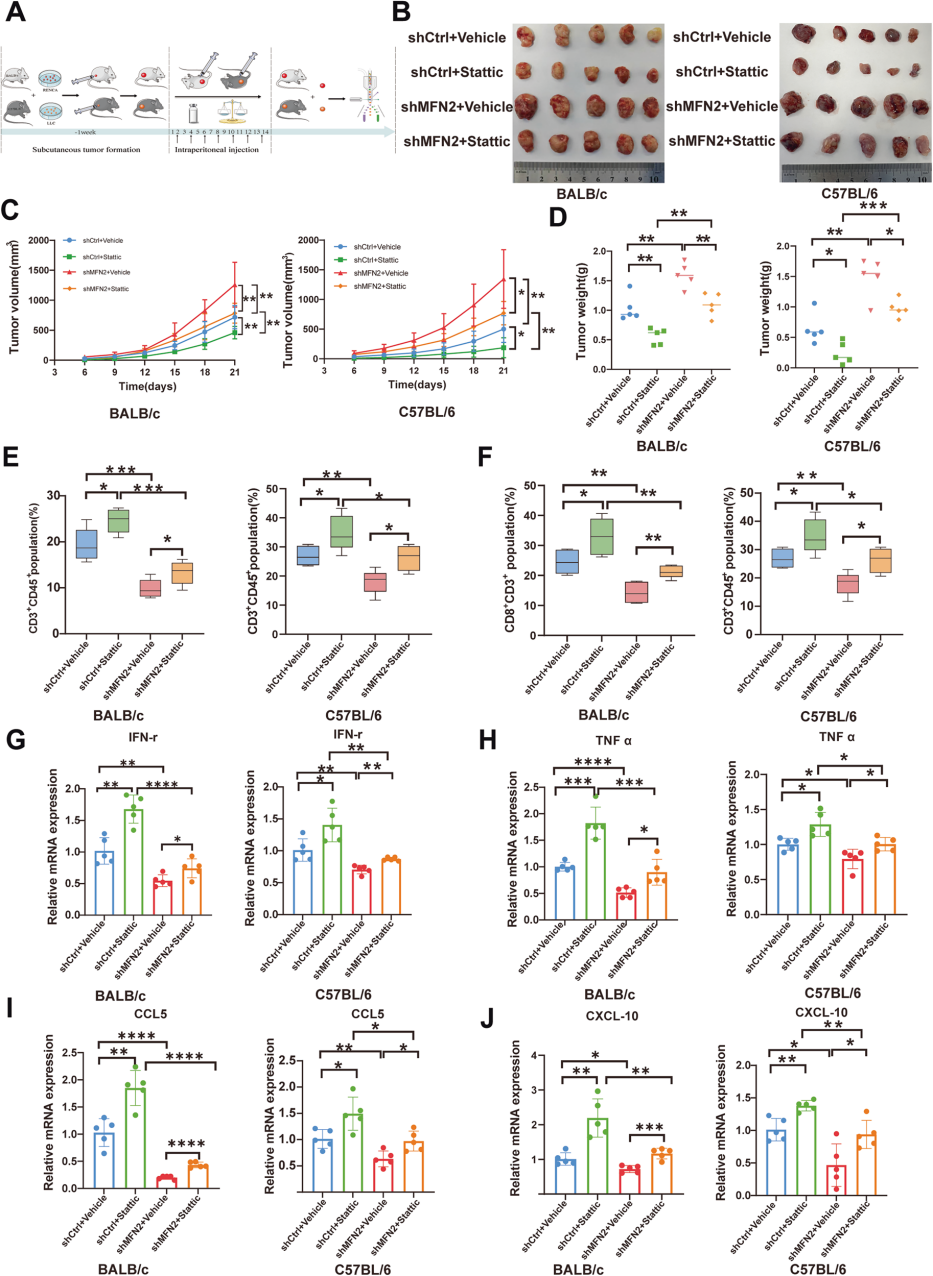
Inhibition of p-STAT3 suppresses MFN2 knockdown–induced tumor growth and immune evasion. **A** Experimental workflow for subcutaneous tumor modeling and pharmacological intervention. **B** Tumor mass of dissected tumors from mice across different intervention groups. **C** Tumor growth curves showing mean tumor volume over time in mice bearing subcutaneous tumors under different treatment regimens, Two-way ANOVA (n = 5). **D** Comparison of tumor weights among different intervention groups in mice, One-way ANOVA followed by Tukey’s test (n = 5). **E** Proportion of CD3^+^CD45^+^ T lymphocytes in tumor tissues from different treatment groups, One-way ANOVA followed by Tukey’s test (n = 5). **F** Proportion of CD8^+^CD3^+^ T lymphocytes in tumor tissues from different treatment groups, One-way ANOVA followed by Tukey’s test. The RT-qPCR analysis of the expressions of IFN-γ (**G**), TNF-α (**H**), CCL-5 (**I**), and CXCL-10 (**J**) in bulk RENCA and LLC tumor xenografts, One-way ANOVA followed by Tukey’s test (n = 5). Data were shown as mean ± SEM; *P < 0.05, **P < 0.01, ***P < 0.001, ****P < 0.0001.

To evaluate the therapeutic potential of targeting the STAT3 pathway in the context of MFN2 deficiency, we established syngeneic subcutaneous xenograft models by injecting LLC and Renca cells into immunocompetent C57BL/6 and BALB/c mice, respectively (Fig. 7A). Once tumors reached approximately 100 mm³, mice were treated with the p-STAT3 inhibitor Stattic or the STAT3 DNA-binding inhibitor S3I-201 via intraperitoneal injection every other day. Notably, no significant body weight loss (Fig. S5A) or systemic toxicity was observed, indicating the safety and tolerability of the treatment. Consistent with our in vitro findings, MFN2 knockdown significantly accelerated tumor kinetics in both models. However, pharmacological inhibition of STAT3 effectively abrogated this accelerated growth (Fig. 7B, C) (Fig. S5B, C) and markedly reduced the final tumor burden (Fig. 7D) (Fig. S5D).

To further explore the underlying immune mechanisms, we characterized the tumor-infiltrating lymphocytes (TILs) via flow cytometry. MFN2 knockdown led to a profound reduction in both CD3⁺ and CD8^+^ T-cell infiltration within the TME (Fig. 7E, F) (Fig. S5E, F). Conversely, STAT3 inhibition successfully restored and even expanded these T-cell populations. This shift in the cellular landscape was accompanied by significant changes in the cytokine/chemokine profile: the expression levels of effector cytokines (IFN-γ, TNFα) and key T-cell-recruiting chemokines (CCL5, CXCL10) were significantly downregulated following MFN2 silencing but were markedly rescued upon STAT3 administration (Fig. 7G–J) (Fig. S5G–J). Collectively, these in vivo results demonstrate that MFN2 deficiency drives a ‘cold’ tumor microenvironment through the STAT3 axis. Importantly, they prove that pharmacological inhibition of STAT3 can effectively counteract the systemic immunosuppression and pro-tumorigenic effects induced by MFN2 loss, highlighting the MFN2/STAT3 axis as a promising therapeutic target.

### Validation of the MFN2/EGFR/STAT3/PD-L1 axis in patient-derived tumor organoids

**Fig. 8:**
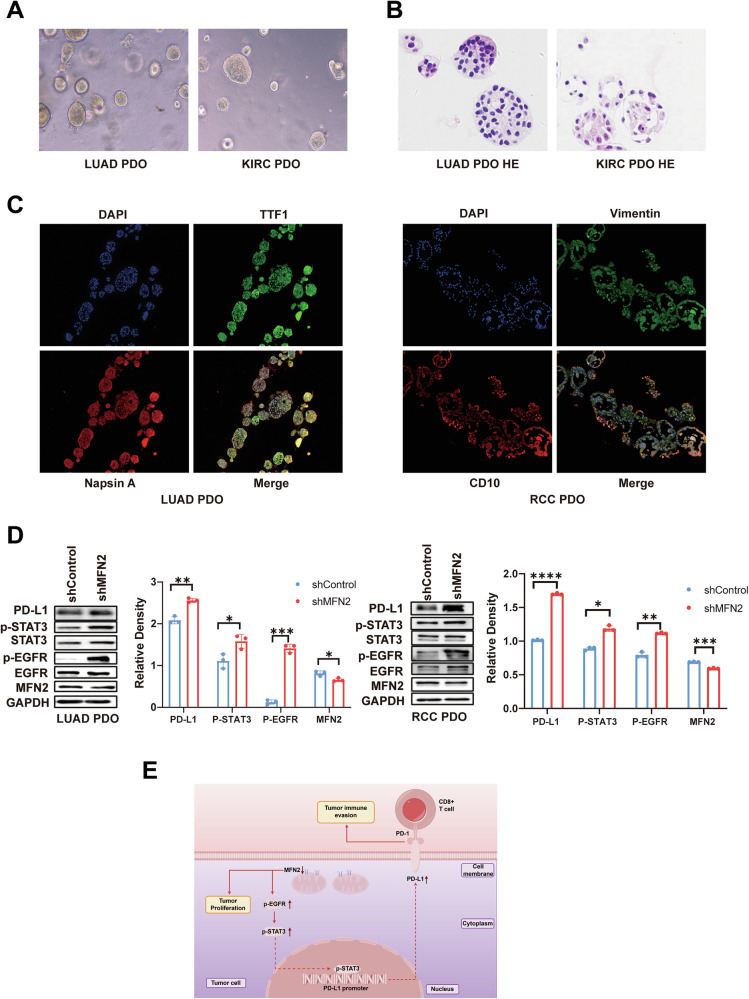
Validation of MFN2-mediated regulation of PD-L1 expression via the EGFR/STAT3 signaling pathway at the tumor organoid level. **A** Bright-field images of LUAD and KIRC organoids. **B** Hematoxylin–eosin (H&E) staining of LUAD and KIRC organoids. **C** Immunofluorescence confocal identification of organoid models derived from human KIRC and LUAD tissues. **D** Western blot analysis of EGFR pathway proteins in MFN2-knockdown KIRC and LUAD organoids using the indicated antibodies, Student’s *t* test (n = 3). **E** A schematic model of MFN2 regulation of PD-L1. Data were shown as mean ± SEM; *P < 0.05, **P < 0.01, ***P < 0.001, ****P < 0.0001.

To further validate the regulatory role of MFN2 in a physiologically relevant context, we established patient-derived organoid (PDO) models of LUAD and KIRC using surgically resected tumor tissues (Fig. 8A). Hematoxylin and eosin (H&E) staining (Fig. 8B) and immunofluorescence analysis of lineage-specific markers (Fig. 8C) confirmed that these PDOs faithfully recapitulated the histological and biological characteristics of their corresponding primary tumors.

Following the successful characterization of the models, we employed lentiviral transduction to stablely knockdown MFN2 expression in both LUAD and KIRC organoids. Consistent with our findings in cell lines, MFN2 deficiency in the organoid models triggered a marked increase in the phosphorylation levels of EGFR (p-EGFR) and STAT3 (p-STAT3). Crucially, this activation of the EGFR/STAT3 signaling cascade was accompanied by a significant upregulation of PD-L1 protein expression (Fig. 8D). These results from patient-derived ex vivo models provide robust evidence that MFN2 serves as a critical suppressor of the EGFR/STAT3/PD-L1 axis in human lung and kidney cancers.

## Discussion

Cancer cells employ diverse immune evasion strategies, among which immunoediting stands as a primary mechanism. This process involves the aberrant modulation of immune checkpoints, such as PD-L1, which paralyzes T-cell activation and expansion, enabling tumors to circumvent immunosurveillance [18]. Despite the clinical breakthroughs of PD-1/PD-L1 blockade, low response rates—driven by heterogeneous PD-L1 expression and acquired resistance—underscore the urgency of unraveling the molecular determinants of PD-L1 stability and regulation.

In this study, we identify the mitochondrial outer-membrane protein Mitofusin-2 (MFN2) as a novel regulator of PD-L1. We demonstrate that MFN2 deficiency facilitates tumor immune evasion by augmenting the EGFR-dependent nuclear translocation of p-STAT3 (Fig. 8E). While MFN2 downregulation has been previously linked to tumor proliferation [15, 22, 30] and our own earlier work highlighted its role in autophagic disruption [20], its contribution to the immune landscape remained elusive. Here, we extend these findings by showing that reduced MFN2 levels in lung adenocarcinoma (LUAD) and kidney renal clear cell carcinoma (KIRC) correlate inversely with PD-L1 elevation. Our results establish MFN2 as a negative regulator of PD-L1-mediated immune escape, suggesting that its loss acts as a dual driver of both metabolic dysfunction and immune subversion.

Our findings provide further insight by demonstrating that reduced MFN2 expression results in the upregulation of PD-L1 across various malignancies, including lung, kidney, and breast cancers. This elevation occurred at both the protein and mRNA levels, indicating that MFN2 regulates PD-L1 primarily through transcriptional mechanisms. By modulating PD-L1 abundance, MFN2 deficiency may play a pivotal role in driving immune escape and conferring resistance to immune checkpoint blockade (ICB) therapies.

The tumor microenvironment (TME) is characterized by a complex interplay of extrinsic stressors, such as hypoxia, energy deprivation, and the presence of pro-inflammatory cytokines like IFN-γ [31]. Our study demonstrates that the downregulation of MFN2 acts as a potent endogenous driver that exacerbates PD-L1 overexpression within this multifaceted milieu.

Specifically, while environmental cues such as energy deficiency [32] or hypoxia [33] trigger PD-L1 expression, MFN2 deficiency provides an additional layer of transcriptional activation by unleashing the EGFR/STAT3 pathway. Notably, in the presence of IFN-γ, the loss of MFN2 exerts a synergistic effect, converging classical STAT1-mediated induction with persistent STAT3 activation [34]. This “double-hit” mechanism significantly elevates the threshold of PD-L1 expression, effectively shielding tumor cells from T-cell-mediated cytotoxicity even under extreme physiological stress. Consequently, the depletion of MFN2 reshapes the TME into a highly immunosuppressive niche, creating a vicious cycle that accelerates tumor progression and immune evasion.

Building on these findings, we demonstrated that MFN2 regulates PD-L1 expression via the EGFR/STAT3 signaling axis. The loss of MFN2 enhanced EGFR phosphorylation and downstream STAT3 activation, both of which are established drivers of PD-L1 transcription and immune escape. Once activated, STAT3 directly bound to the CD274 promoter, effectively bypassing post-translational regulatory checkpoints; accordingly, the observed changes in PD-L1 mRNA levels, total protein abundance, and cell-surface localization were highly consistent [35–37]. Importantly, pharmacological inhibition of STAT3 completely abrogated the PD-L1 upregulation induced by MFN2 knockdown. These results underscore the central role of the STAT3 pathway in MFN2-mediated immune modulation and highlight its potential as a therapeutic target in tumors characterized by MFN2 deficiency and elevated PD-L1 expression.

These findings offer important clinical implications, particularly for immune checkpoint blockade therapies targeting PD-1/PD-L1. Despite their success, resistance remains a significant barrier, often due to the compensatory upregulation of PD-L1. Our study suggests that targeting MFN2, or the EGFR/STAT3 pathway, may help overcome this resistance. By preventing PD-L1 upregulation, this approach could enhance the efficacy of PD-1/PD-L1 blockade therapies, thereby improving patient outcomes in cancers with high PD-L1 expression.

Previous studies have shown that mitochondrial fusion protein 2 (MFN2) in CD8^+^ T cells regulates their metabolic fitness and cytotoxic function [38], indicating that T cell–intrinsic MFN2 can shape the tumor immune microenvironment (TIME) by modulating CD8^+^ T-cell function. However, this work has largely focused on how MFN2 in CD8^+^ T cells affects their cytotoxicity and thereby influences the tumor microenvironment. In contrast, our study is the first to elucidate the regulatory role of MFN2 in PD-L1 expression within tumor cells, which consequently suppresses the cytotoxic activity of CD8^+^ T cells and remodels the tumor microenvironment. Consistent with this mechanism, T-cell killing assays and single-cell analyses of lung cancer tissues show that tumor-cell MFN2 expression influences both the cytolytic capacity and infiltration of CD8^+^ T cells. In xenograft models, MFN2 knockdown accelerated tumor growth and reduced CD8^+^ T-cell infiltration, indicative of an immunosuppressive phenotype. Notably, inhibition of STAT3 reversed these effects, suggesting that combining STAT3 inhibitors with PD-1/PD-L1 immunotherapy may be effective. Collectively, these findings support combination strategies targeting the PD-1/PD-L1 axis together with the EGFR/STAT3 pathway to enhance therapeutic efficacy and overcome immune evasion.

The orchestration of mitochondrial dynamics by MFN2 provides a critical regulatory link between cellular metabolism and systemic antitumor immunity. In this study, we demonstrate that MFN2 deficiency does not merely upregulate PD-L1 in isolation but appears to trigger a multimodal immunosuppressive program that reshapes the tumor microenvironment (TME) into a “cold” or “immune-desert” phenotype.

Our data suggest that MFN2 loss may facilitate a “dual-escape” strategy to evade both innate and adaptive immune surveillance. While the upregulation of PD-L1 provides a molecular shield against T-cell-mediated cytotoxicity (adaptive immunity), we also observed a concurrent elevation of CD47—the critical “don’t eat me” signal—which is known to thwart macrophage-mediated phagocytosis (innate immunity). Although the precise regulatory mechanism and functional consequence of the MFN2-CD47 axis warrant further experimental validation, this synergistic co-regulation positions MFN2 as a potential molecular gatekeeper. Its depletion may provide tumors with a comprehensive, multi-layered defense mechanism, effectively shielding them from the host’s integrated immune response.

Beyond mechanistic insights, our findings emphasize the critical role of MFN2 as a negative regulator of PD-L1-mediated immune evasion, exerting a dual impact on both immune checkpoint expression and T-cell effector functions. These results position MFN2 as a promising prognostic biomarker and a strategic therapeutic target, particularly in malignancies characterized by aberrant EGFR/STAT3 signaling and PD-L1 expression. Future investigations should prioritize the efficacy of targeting the MFN2 axis, potentially through combination therapies with EGFR/STAT3 pathway inhibitors, to sensitize “cold” tumors and overcome resistance to current PD-1/PD-L1 blockade. Such an integrated approach holds the potential to break the metabolic-immune deadlock and optimize treatment outcomes for patients with recalcitrant malignancies.

In conclusion, our research provides a comprehensive understanding of the molecular mechanisms driving immune evasion by identifying MFN2 as a key determinant of PD-L1 transcription. By clarifying the interplay between mitochondrial dynamics and checkpoint signaling, we have uncovered a new vulnerability in tumor-associated immunosuppression. Pharmacological modulation of the MFN2-EGFR/STAT3 axis may represent a potent strategy to synergize with existing immunotherapies, ultimately overcoming resistance and improving long-term outcomes for patients with advanced cancer.

## Materials and methods

### Clinical samples

We retrospectively included 50 patients with lung cancer and 50 patients with renal cancer who underwent curative resection at the First Affiliated Hospital of Shandong Second Medical University between 2020 and 2025. All participants provided written informed consent for the use of their clinical specimens. None of the patients received anti-tumor therapy prior to surgery. Tumor specimens were obtained from formalin-fixed paraffin-embedded tissues for immunohistochemical analysis. All available histological sections were reviewed by pathologists specializing in respiratory and urinary system diseases. Additionally, six fresh tissue samples from patients with lung adenocarcinoma (LUAD) and kidney renal clear cell carcinoma (KIRC), including adjacent non-tumor and tumor tissues, were used for protein expression analysis, and three tumor specimens were employed for organoid culture. Clinical information is provided in Supplementary Tables S3 and S4. All procedures were conducted in accordance with the Declaration of Helsinki. Informed consent was obtained from all participants prior to sample collection. This study was approved by the Ethics Committee of the First Affiliated Hospital of Shandong Second Medical University(Approval No. KYLL20250709-2).

### Cell lines

The human lung cancer cell lines A549 (CCL-185, RRID:CVCL_0023), NCI-H1299(CRL-5803, RRID:CVCL_0060), HCC827(CRL-2868, RRID:CVCL_2063), NCI-H1703(CRL-5889,RRID:CVCL_1490) and NCI-H1975 (CRL-5908, RRID:CVCL_1511), the human kidney cell line 786-O(CRL-1932, RRID:CVCL_1051), 769-P(CRL-1933, RRID:CVCL_1050) and the human breast cancer cell line MDA-MB-231(CRM-HTB-26, RRID:CVCL_0062) were purchased from ATCC (USA). OS-RC-2(CL-0177, RRID:CVCL_1626) and PC-9(CL-0668, RRID:CVCL_B260) were purchased from Procell (Wuhan, China), the mouse lung cancer cell line LL/2 (LLC1)(C5094, RRID: CVCL_4358) and the mouse kidney cancer cell line RENCA (C5190; RRID:CVCL_2174) were purchased from the BDBIO (Hangzhou, China). All cells were tested for mycoplasma contamination and authenticated using short tandem repeat fingerprinting before use.

### RNA sequencing data

Total RNA was extracted from 786-O and A549 cells with stable MFN2 knockdown, as well as from control cells, using TRIzol Reagent (Life Technologies, USA). RNA purity and concentration were determined using a NanoDrop 2000 spectrophotometer (Thermo Fisher Scientific, USA), and RNA integrity was assessed with the RNA Nano 6000 Assay Kit on the Agilent Bioanalyzer 2100 system (Agilent Technologies, USA). For transcriptome sequencing, 1 μg of high-quality total RNA per sample was used to construct libraries using the Hieff NGS Ultima Dual-mode mRNA Library Prep Kit for Illumina (Yeasen Biotechnology, China). Poly-T oligo-attached magnetic beads were used to enrich mRNA, followed by first- and second-strand cDNA synthesis, end repair, adaptor ligation, and PCR amplification. Purified libraries were sequenced on an Illumina NovaSeq platform (Illumina, USA) to generate 150 bp paired-end reads. Raw reads were processed using the BMKCloud platform (www.biocloud.net) to remove adapter sequences, poly-N, and low-quality reads. Clean reads were aligned to the reference genome using HISAT2 (v2.1.0), and only reads with perfect matches or one mismatch were retained for further analysis. Differential gene expression was evaluated using fold change and p-value thresholds.

### Extraction of nuclear and cytoplasmic proteins

Cytoplasmic and nuclear protein fractions were isolated using the Nuclear Protein Extraction Kit (Solarbio, Cat. No. R0050, China), following the manufacturer’s protocol.

### Cell culture, lentiviral transduction, and gene silencing

A549, MDA-MB-231, PC-9, and RENCA cells were cultured in DMEM supplemented with 10% fetal bovine serum (FBS), while 786-O, H1299, HCC827, H1703, H1975, 769-P, and LLC cells were maintained in RPMI-1640 medium containing 10% FBS. All cultures were incubated at 37 °C in a humidified atmosphere with 5% CO₂. Prior to experimentation, all cell lines were authenticated by short tandem repeat (STR) profiling and confirmed to be free of mycoplasma contamination. Lentiviral particles were produced in HEK-293T cells using VSVG and psPAX2 packaging plasmids. Supernatants were harvested at 48 and 72 h post-transfection and used to infect A549 cells, followed sequentially by 786-O, RENCA, and LLC cells. Stable MFN2-knockout cell lines were established by puromycin selection (2 μg/mL) and validated by RT-qPCR. For transient gene silencing, MFN2 and STAT3 siRNAs (GenePharma, China) were transfected using Lipofectamine 2000 (Invitrogen, USA) in serum-free DMEM or RPMI-1640 medium at a final concentration of 50 nM. After 6 h, the medium was replaced with complete growth medium, and cells were cultured for an additional 24 or 36 h. Knockdown efficiency was confirmed by western blotting. The sequences of all siRNAs used are listed in Supplementary Table S2.

### Western blot

After the cells were cultured for the indicated times, total protein was collected to perform the SDS polyacrylamide gel electrophoresis as described in our previous study [17]. The antibodies used for western blot were shown in supplementary file -Table S1.

### Enzyme-linked immunosorbent assay (ELISA)

The concentrations of TNF-α(JL10208), IFN-γ (JL12152) were assessed using ELISA Kits (all from JONLNBIO, China) according to the kit instructions.

### Bioinformatics analysis

MFN2 expression was analyzed using the TNMplot web tool (Tumor/Normal/Metastatic module; https://tnmplot.com/analysis), and between-group differences were evaluated with the Mann–Whitney U test [39]. Overall survival (OS) analyses for MFN2 were generated with the Kaplan–Meier Plotter using the lung and kidney cancer datasets (https://kmplot.com/analysis) [40, 41].

### Analyses of TCGA data

Clinical and RNA-seq data for the TCGA Kidney Renal Clear Cell Carcinoma (TCGA-KIRC) and Lung Adenocarcinoma (TCGA-LUAD) cohorts were obtained from FireBrowse (http://firebrowse.org/). Normalized protein expression data for the same cohorts were downloaded from the Clinical Proteomic Tumor Analysis Consortium (CPTAC) data portal (https://pdc.cancer.gov/pdc/browse). Differential expression analyses were performed in R using DESeq2 (v1.30.1). We computed fold changes and adjusted P values for MFN2 expression by comparing tumor with adjacent normal tissues in KIRC and LUAD. DESeq2 (v1.30.1) was also used to identify differentially expressed genes (DEGs) between normal and tumor tissues, as well as between MFN2-high (>50%) and MFN2-low (≤50%) tumors. Receiver-operating characteristic analyses were conducted with pROC (v1.18.0) to evaluate the prognostic performance (area under the curve, AUC) of candidate genes. Heatmaps were generated with pheatmap (v1.0.12) using row-scaled expression values, and chord diagrams were produced with ggplot2 (v3.0.0).

### Data acquisition, quality control, data integration, dimensionality reduction, and clustering

Single-cell RNA sequencing data used in this study were obtained from GEO database (accession number: GSE189357), which includes 8 lung cancer samples, consisting of 33538 genes and 122373 cells in total. Basic quality control (QC) was performed to ensure accuracy and reliability in the analysis. The quality control criteria were as follows: nFeatureRNA was between 180 and 6000, mitochondrial gene expression percentage (percent_mito) was less than 20%, and each gene was expressed in at least three cells. After applying these standards, a total of 25041 genes and 115242 cells were retained for further analysis. We integrated the filtered count matrices from 8 samples using the R package harmony (v1.0) approach to correct for batch effects and integration. After integration, principal component analysis (PCA) was performed on the integrated data followed by embedding into low-dimensional space with Uniform Manifold Approximation and Projection (UMAP) based on the top 30 dimensions. Clusters were generated by graph-based method using the FindClusters function from the Seurat package (v4.3.0).

To investigate the correlation between MFN2 expression levels in epithelial cells and immune infiltration, we quantified MFN2 expression within the epithelial cell population. Epithelial cells were stratified into MFN2-high and MFN2-low subgroups using the median MFN2 expression level across all epithelial cells as the cut-off. Additionally, we calculated the average MFN2 expression level of epithelial cells for each individual sample. Using the median of these sample-wise average values as the cut-off, samples were categorized into MFN2-high and MFN2-low groups. The correlation between MFN2 expression status and immune infiltration characteristics was subsequently analyzed for both the cell-level and sample-level groupings.

### Flow cytometry and analysis

To analyze membrane PD-L1 expression using flow cytometry, A549, 786-O, or 769-P cells were harvested by centrifugation at 1000 × *g* for 5 min and incubated with PBS containing 0.5% bovine serum albumin for 10 min at room temperature. Cells were then stained with a PD-L1-FITC MFI antibody and an isotype-matched control at 4 °C for 30 min in the dark. After washing three times with PBS, cells were analyzed on a BD FACSAria II flow cytometer. Data were processed using FlowJo X software, and PD-L1 membrane expression levels were assessed based on the median fluorescence intensity (MFI) of PD-L1-FITC.

For murine tumor samples, single-cell suspensions (~1 × 10⁶ cells per sample) were prepared and subjected to immunophenotyping. Cells were first incubated with fluorochrome-conjugated antibodies against CD45, CD3, CD4, and CD8a at 4 °C in the dark for 30 min. To exclude dead cells, DAPI staining was performed at room temperature in the dark for 5 min. After staining, cells were centrifuged and resuspended in 300 μL of FACS buffer. Flow cytometric analysis was carried out using a BD flow cytometer, and data were analyzed with FlowJo X software. Details of the antibodies used are provided in Supplementary Table S1.

### Immunofluorescence

The cells were cultured overnight at room temperature on chamber slides, then fixed with 4% formaldehyde in PBS for 10 min. This was followed by permeabilization with 0.1% Triton X-100 in PBS for 10 min. Afterward, the cells were blocked with 1% BSA in PBS at room temperature for 30 min and incubated with the specified primary antibody at 4 °C overnight. Subsequently, cells were incubated with an anti-rabbit (or mouse) IgG (H + L), F(ab’)₂ fragment conjugated with Alexa Fluor 594 or 488 at room temperature for 30 min. Coverslips were mounted with an anti-fade medium containing DAPI. Immunofluorescence images were captured using a STELLARIS 5 super-resolution confocal laser scanning microscope (Leica). For each fluorescence channel, all images were acquired using identical settings. Image acquisition and processing were conducted using Leica Application Suite X (Leica).

### Chromatin immunoprecipitation (ChIP)-qPCR

ChIP experiments were performed using the Pierce™ Agarose ChIP Kit (26156; Thermo Fisher Scientific, USA) following the manufacturer’s instructions. Briefly, 293 T cells were crosslinked with 1% formaldehyde at room temperature for 30 min to fix the chromatin, followed by sonication to shear the chromatin into fragments ranging from 100 to 500 bp. Immunoprecipitation was then carried out using antibodies against phosphorylated STAT3 (P-STAT3) as the target, with IgG (ab1791, Abcam, UK) used as the negative control. The extracted chromatin was analyzed by qPCR, and the results were normalized to the input. Primer sequences used for ChIP analysis are listed in the Supplemental Table.

### Coimmunoprecipitation (CoIP)

IP buffer supplemented with protease inhibitor cocktail and phosphatase inhibitor cocktail was used to extract proteins from cells. After the concentration was measured by the BCA method, the cell lysates were divided into three parts and then incubated with the 2.5 µL of MFN2 (12186-1-AP Proteintech) or 0.8 µL of normal rabbit IgG (98136-1-RR, Proteintech) at 4 °C overnight. 60 µL of Protein A/G Magnetic Beads (HY-K0202, MCE) were added to the incubated protein and incubated 4-6 h at 4 °C.Then, the magnetic beads were washed with IP buffer, and the conjugated protein was finally dissolved in 80 µL of 1× protein loading buffer (Solarbio, Beijing, China) and boiled at 100 °C for 10 min for Western Blot.

### Immunohistochemistry staining

Formalin-fixed, paraffin-embedded tissue sections were deparaffinized in xylene, rehydrated through a graded ethanol series, and subjected to heat-induced antigen retrieval in citrate buffer (100 °C for 15 min). Primary antibodies diluted in 3% bovine serum albumin (BSA) were applied to the sections and incubated overnight at 4 °C. Following rinsing with phosphate-buffered saline (PBS), the slides were incubated at room temperature for 60 min with horseradish peroxidase (HRP)-conjugated secondary antibodies (anti-rabbit or anti-mouse), also diluted in 3% BSA. Sections were then dehydrated through an ascending ethanol gradient (50%, 70%, 80%, 95%, and 100%) and mounted using a stabilizing medium. Images were captured using a MoticEasyScan One digital slide scanner under standard acquisition settings. Immunohistochemical (IHC) staining density was quantified using ImageJ (Fiji v1.51j) by measuring both the average staining intensity and the proportion of positively stained cells. A composite protein abundance score was subsequently calculated from these metrics.

### Xenograft mouse model

In the subcutaneous xenograft experiment, 1 × 10⁶ Renca or LLC cells (sh-MFN2 or sh-control) were suspended in 100 μL of PBS and inoculated into the lateral abdominal wall of BALB/c and C57BL/6 mice, respectively. When tumors reached approximately 100 mm³ in volume, Stattic (HY-13818, MCE, USA) was administered intraperitoneally every other day at a dose of 10 mg/kg. Tumor dimensions were measured every 2–3 days, and tumor volumes were calculated using the formula: ½ × (length × width²). Tumor weights were recorded upon termination. Mice were euthanized following the guidelines outlined in the *AVMA Guidelines for the Euthanasia of Animals* (2020). All procedures were approved by the Experimental Animal Welfare and Ethics Committee of Shandong Second Medical University (Approval No.: 2025SDL759).

### qRT-PCR assay

Total RNA was extracted using TRIzol reagent (Invitrogen, USA, Cat#15596026), and reverse transcription was subsequently performed according to the manufacturer’s instructions using either the undefined HiScript III 1st Strand cDNA Synthesis Kit (Vazyme, China, Cat#R312-01). The resulting cDNA was analyzed by quantitative real-time PCR using the SupRealQ Ultra Hunter SYBR qPCR Master Mix (Vazyme, China, Cat#Q713-02) to quantify target gene expression levels. Data were analyzed using the 2^^–ΔΔCT^ method and normalized to GAPDH expression. The qRT-PCR primer sequences are listed in Supplementary Table S2.

### CD8^+^ T-cell-mediated tumor cell-killing assays

Peripheral blood mononuclear cells (PBMCs) were isolated from the peripheral blood of healthy donors using Ficoll density gradient centrifugation. CD8^+^ T cells were positively selected from PBMCs using the CD8 MicroBeads Kit (Miltenyi Biotec, Germany, Cat#130-045-201). The purified CD8^+^ T cells were cultured in RPMI 1640 medium supplemented with 10% fetal bovine serum, 1% antibiotics, 1× MEM non-essential amino acids, 1 mM sodium pyruvate, 10 mM HEPES buffer, 20 ng/mL interleukin-2 (IL-2), and CD3/CD28 T-cell activator for two weeks. Following 48 h of plasmid transfection, tumor cells were cocultured with CD8^+^ T cells at a 3-5:1 ratio (T cells: tumor cells) for 48 h. Non-adherent T cells and debris were removed by PBS washing, and viable tumor cells were quantified using crystal violet staining.

### Establishment of LUAD and KIRC patient-derived organoid models

Patient-derived organoid (PDO) models of lung adenocarcinoma and clear cell renal cell carcinoma were established from surgically resected tumor specimens, using protocols adapted from published methods with tumor-specific optimizations. Fresh tumor tissues were collected from treatment-naïve patients immediately following surgical resection and transported on ice to the laboratory within 1 h in ice-cold DMEM/F-12 medium (Gibco, USA, Cat#11320033) supplemented with 50 U/mL penicillin and 50 μg/mL streptomycin. Tissues were washed three times in cold DMEM/F-12 containing antibiotics and mechanically minced into ~1 mm³ fragments using sterile surgical scalpels under aseptic conditions. The fragments were enzymatically digested in DMEM supplemented with 1 mg/mL collagenase V (Sigma-Aldrich, USA, Cat#C9263) for 1 h at 37 °C with gentle agitation. Following digestion, the suspension was filtered through a 100-μm cell strainer to remove debris and undigested tissue. The resulting pellet was washed with PBS and subjected to further digestion using TrypLE Express (Thermo Fisher Scientific, USA, Cat# 12604013) for 5 min at 37 °C to obtain a single-cell suspension. Cells were then centrifuged at 300 × *g* for 5 min at 4 °C.

Cell viability was assessed using trypan blue exclusion. A total of approximately 30,000–100,000 viable cells were resuspended in 50 μL of basal medium and mixed with 100 μL of ice-cold growth factor-reduced Matrigel (Corning, USA, Cat# 356231). Matrigel domes were plated into the center of each well in pre-warmed 24-well culture plates and incubated at 37 °C for 10–15 min to allow solidification. Subsequently, 500 μL of lung adenocarcinoma organoid culture medium (BioGenous, Hangzhou, China, Cat#K2138-LA) and kidney cancer organ culture medium (BioGenous, Hangzhou, China, Cat#K2171-KC) was gently added to each well.

## Supplementary information

CDDIS-25-6343_Original data

FigureS1

FigureS2

FigureS3

FigureS4

FigureS5

FigureS6

CDDIS-25-6343_Supplementary Figure

CDDIS-25-6343_Supplementary Table

## Data Availability

The raw RNA sequencing data (786-O and A549) are available at the National Center for Biotechnology Information (NCBI) database (accession numbers PRJNA1327665 and PRJNA1328631). All other data that support the findings of this study are available from the corresponding author upon reasonable request.
