# Supplementary material for: Mitofusin-2 suppresses tumor immune escape through EGFR/STAT3-mediated PD-L1 transcription
Source: Cell Death Dis. 2026 Mar 27;17(1):364. doi: 10.1038/s41419-026-08668-3 (PMC13039860; doi:10.1038/s41419-026-08668-3)
Supplement: Supplementary file 1 — CDDIS-25-6343_Original data [file 41419_2026_8668_MOESM1_ESM.pdf]

Figure1E

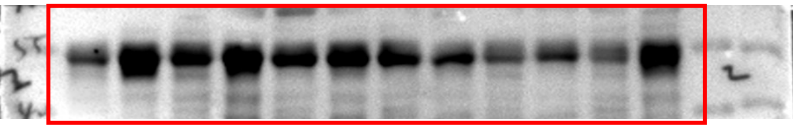

KIRC PD-L1

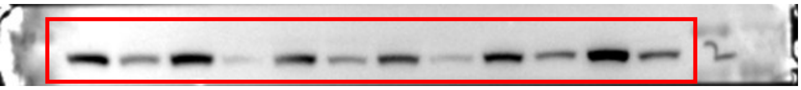

KIRC MFN2

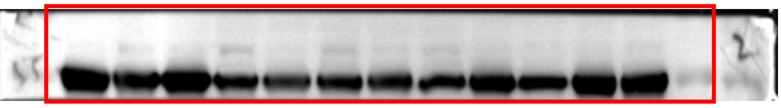

KIRC Tubulin

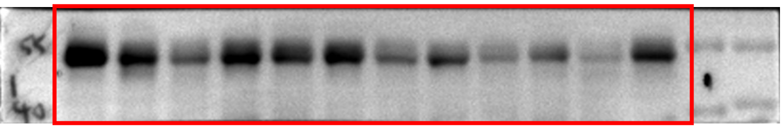

LUAD PD-L1

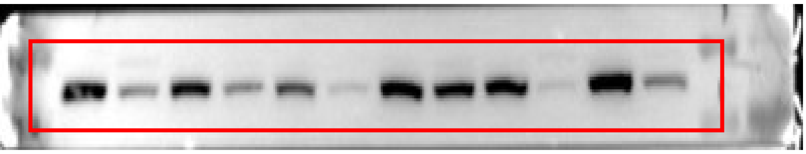

LUAD MFN2

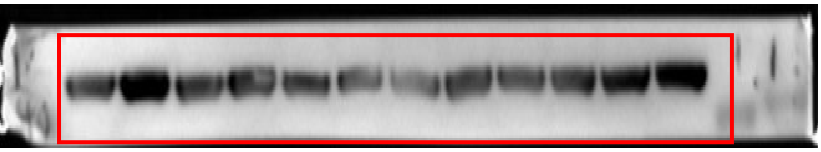

LUAD β-actin

Figure2A

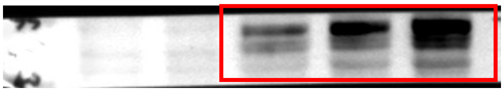

786-O-PD-L1

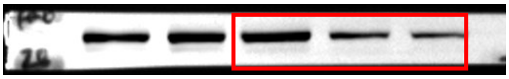

786-O-MFN2

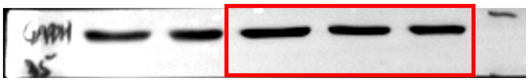

786-O-GAPDH

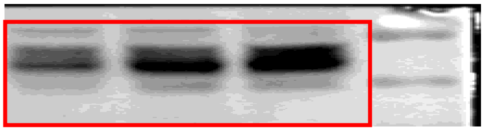

A549-PD-L1

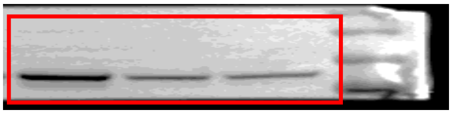

A549-MFN2

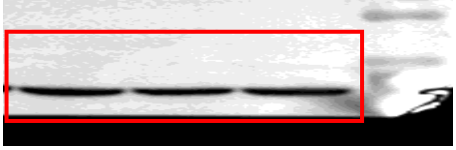

A549-GAPDH

Figure2C

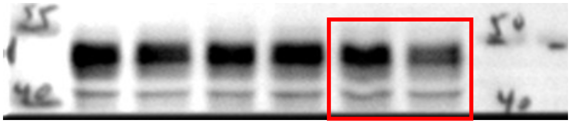

OS-RC-2 PD-L1

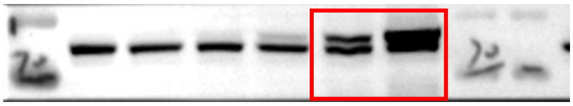

OS-RC-2 MFN2

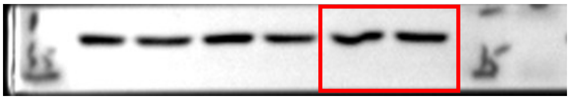

OS-RC-2 GAPDH

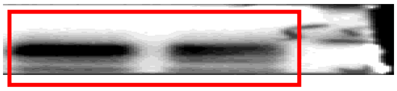

H1975 PD-L1

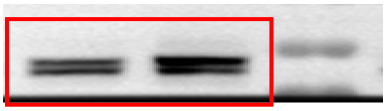

H1975 MFN2

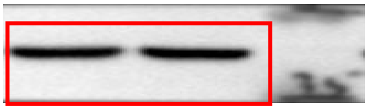

H1975 GAPDH

Figure2D

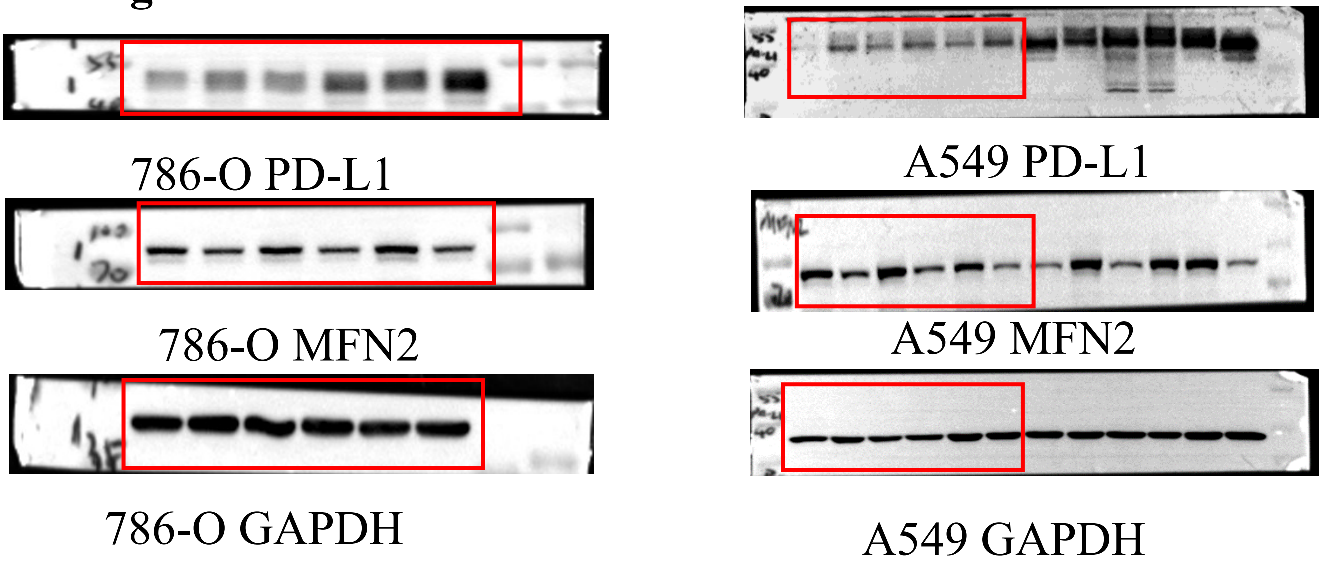

Figure2E

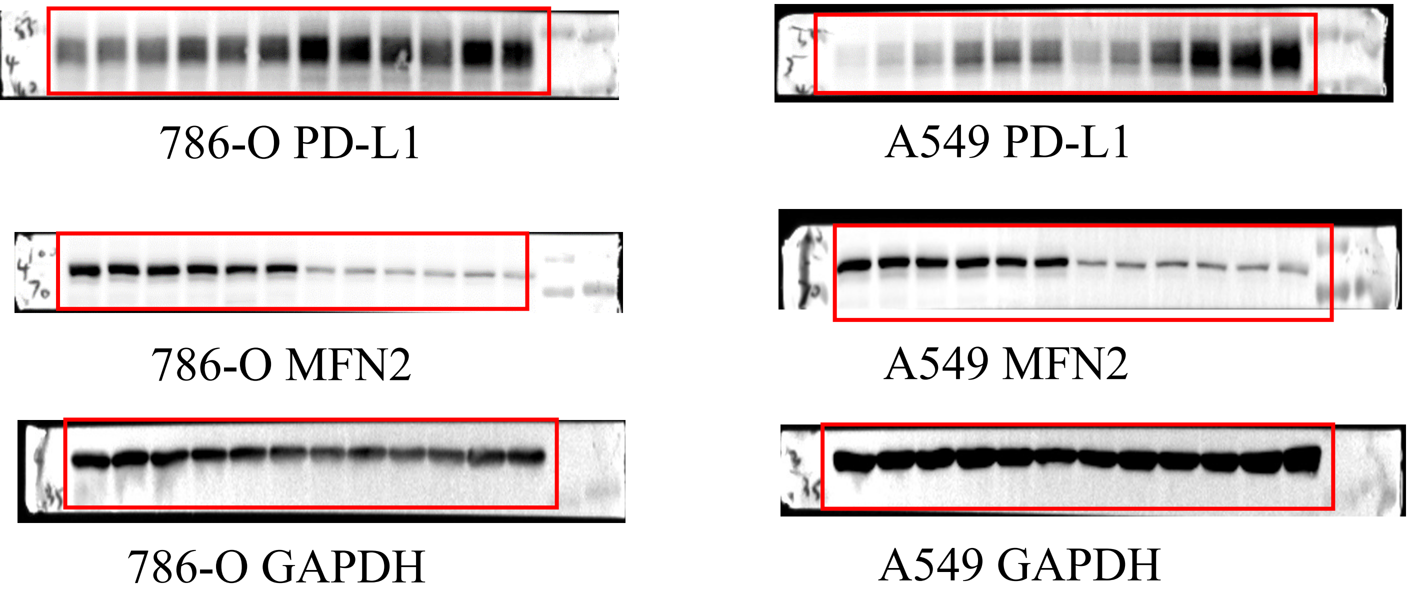

Figure5E

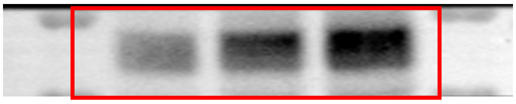

786-O PD-L1

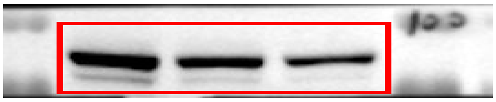

786-O MFN2

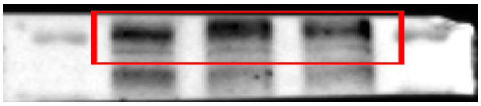

786-O P-EGFR (Y1068)

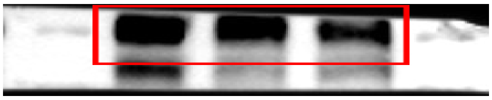

786-O EGFR

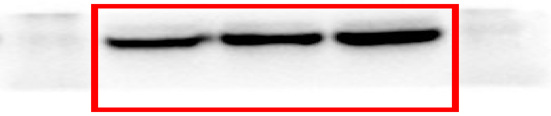

786-O P-STAT3(Y705)

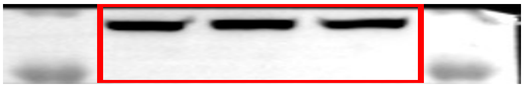

786-O STAT3

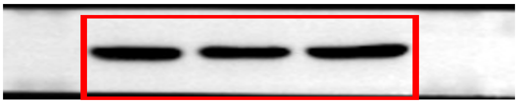

786-O GAPDH

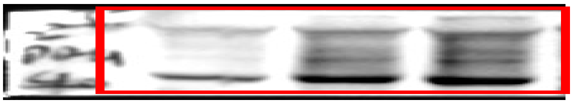

A549 PD-L1

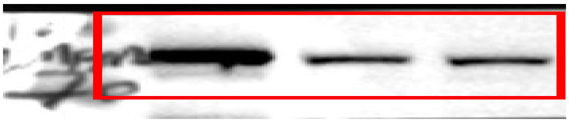

A549 MFN2

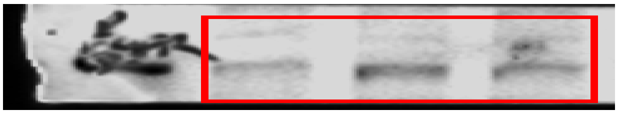

A549 P-EGFR (Y1068)

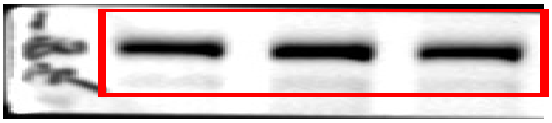

A549 EGFR

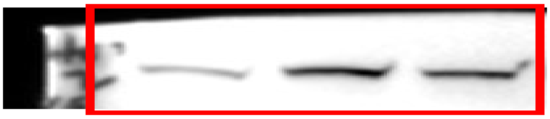

A549 P-STAT3(Y705)

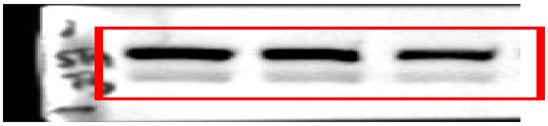

A549 STAT3

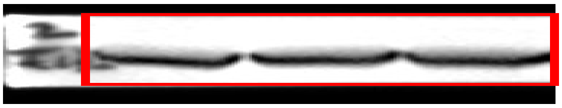

A549 Tubulin

Figure5F

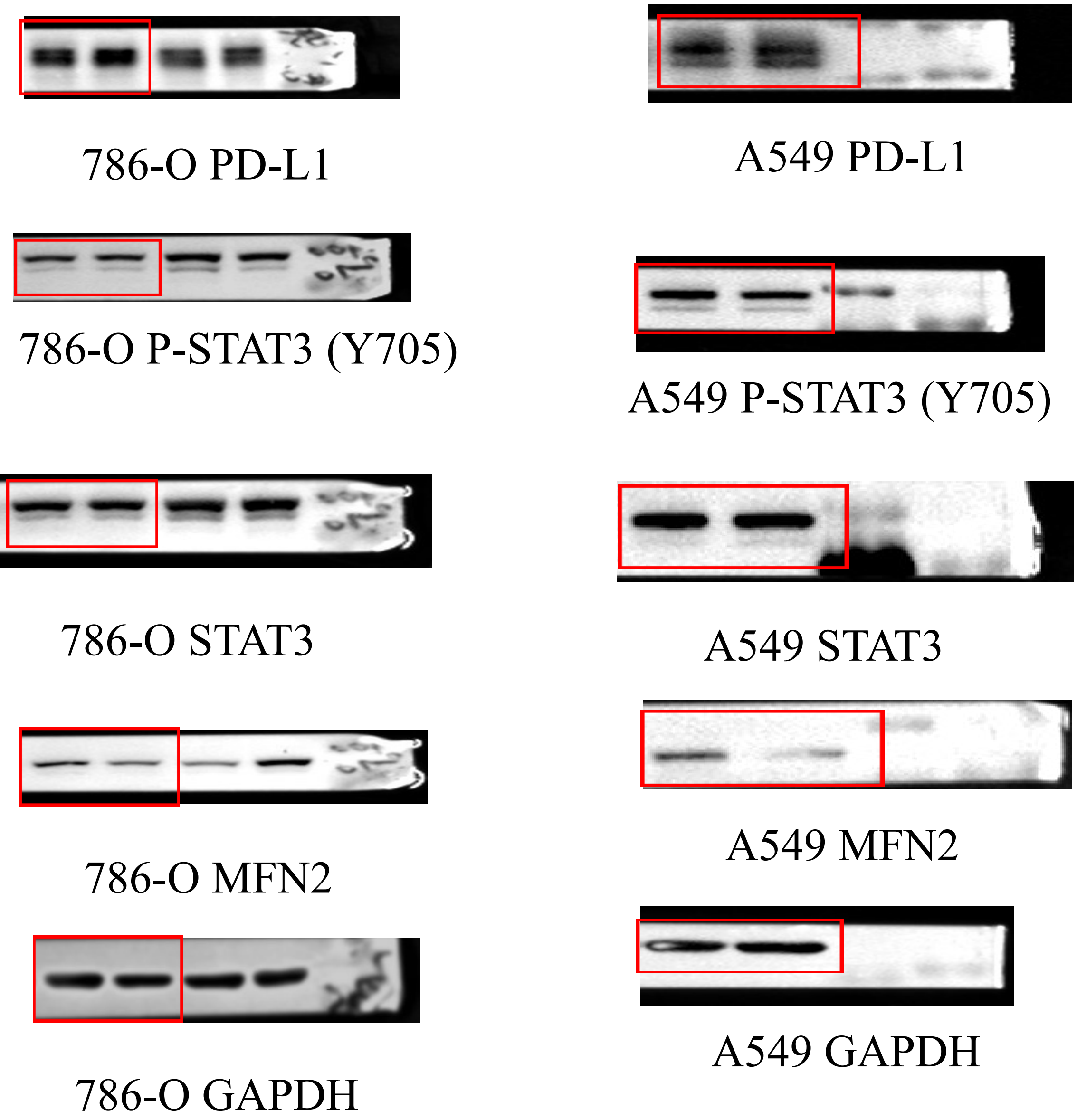
786-O PD-L1
A549 PD-L1
786-O P-STAT3 (Y705)
A549 P-STAT3 (Y705)
786-O STAT3
A549 STAT3
786-O MFN2
A549 MFN2
786-O GAPDH
A549 GAPDH

Figur6A

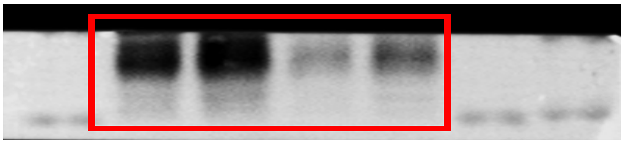

786-O PD-L1

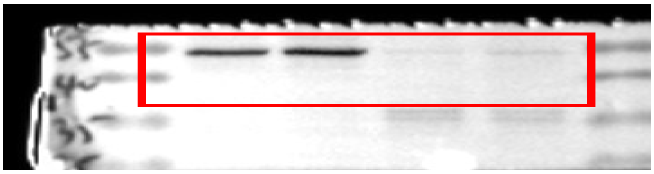

A549 PD-L1

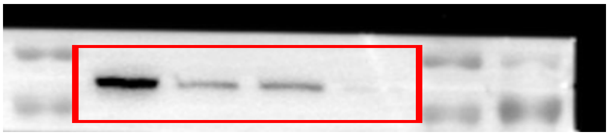

786-O MFN2

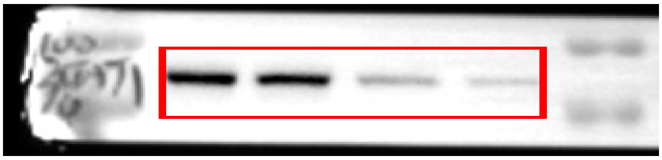

A549 MFN2

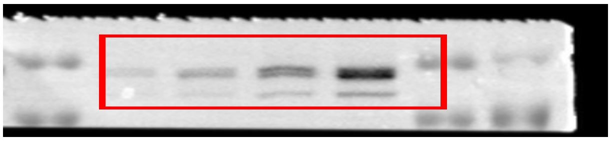

786-O P-STAT3(Y705)

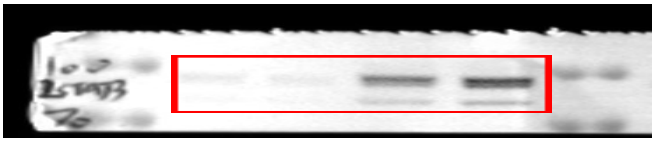

A549 P-STAT3(Y705)

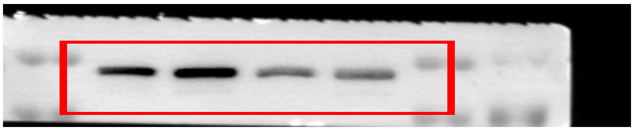

786-O STAT3

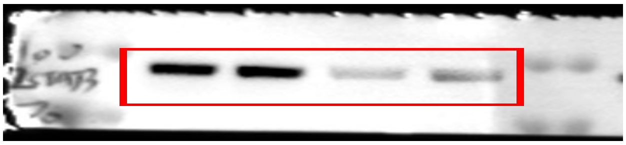

A549 STAT3

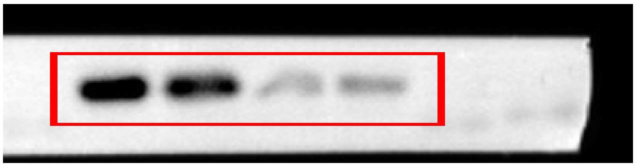

786-O GAPDH

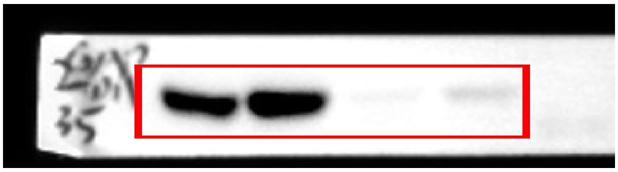

A549 GAPDH

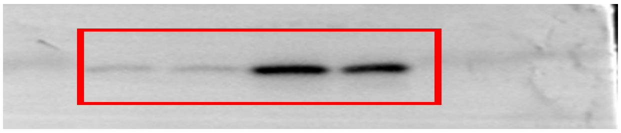

786-O Histone H3

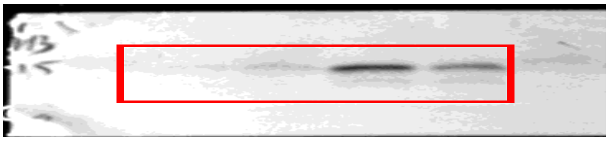

A549 Histone H3

Figur6B

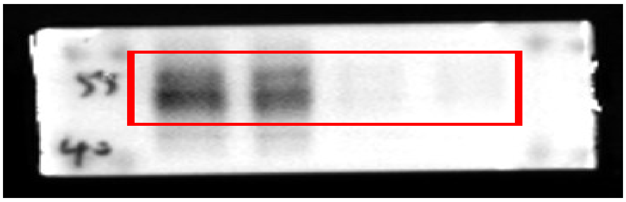

786-O PD-L1

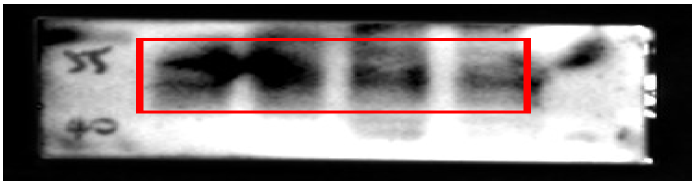

A549 PD-L1

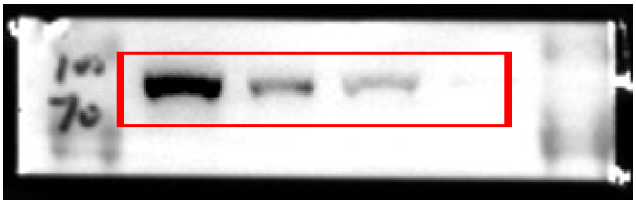

786-O MFN2

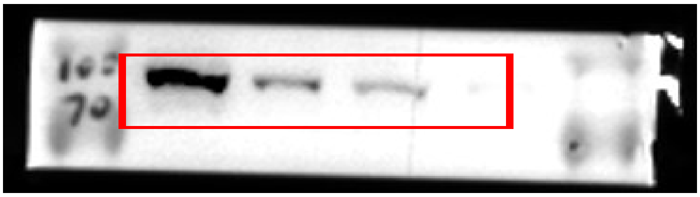

A549 MFN2

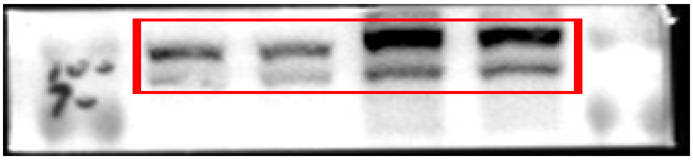

786-O P-STAT3

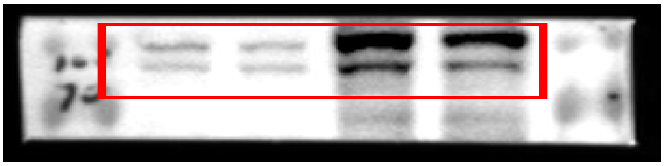

A549 P-STAT3

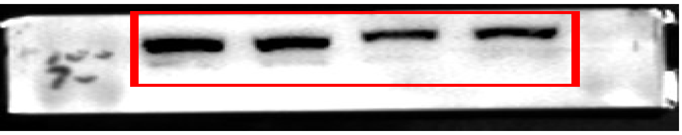

786-O STAT3

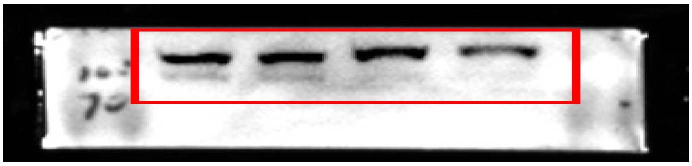

A549 STAT3

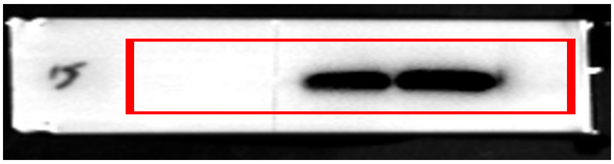

786-O Histone H3

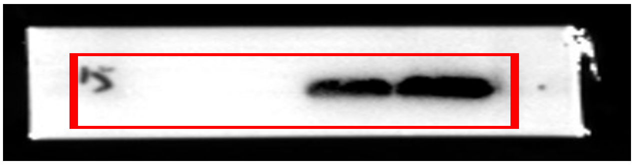

A549 Histone H3

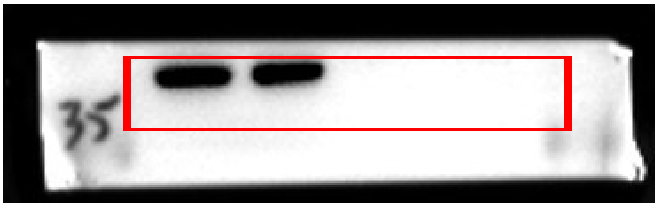

786-O GAPDH

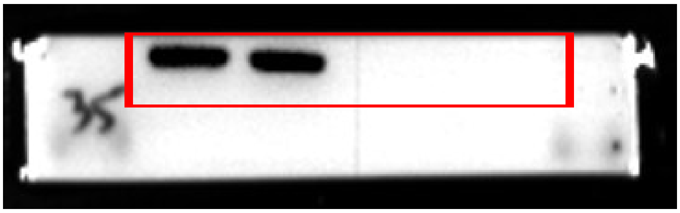

A549 GAPDH

Figure6D

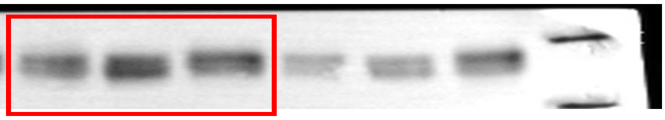

786-O PD-L1

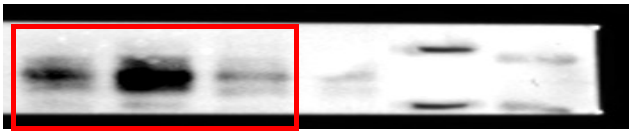

A549 PD-L1

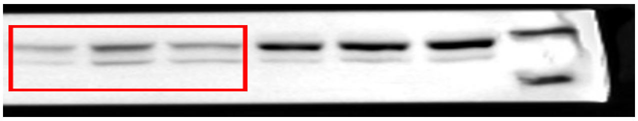

786-O P-STAT3(Y705)

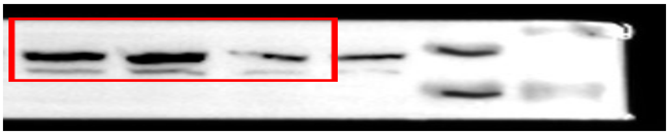

A549 P-STAT3(Y705)

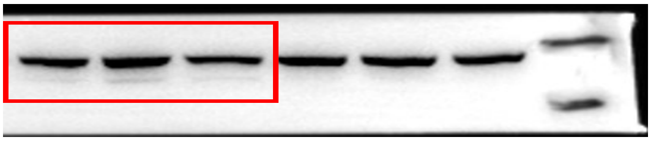

786-O STAT3

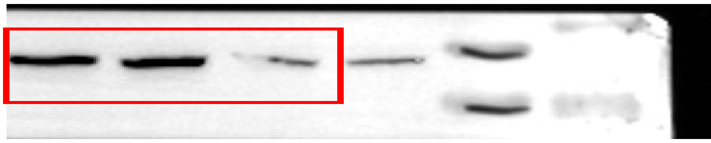

A549 STAT3

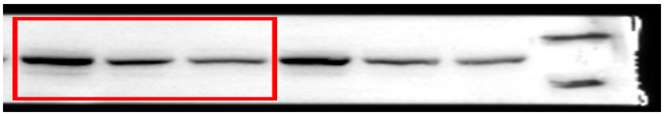

786-O MFN2

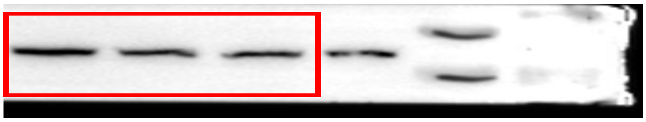

A549 MFN2

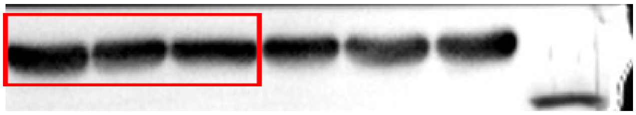

786-O GAPDH

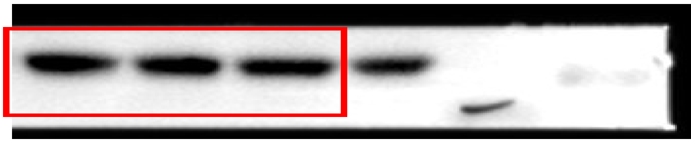

A549 GAPDH

Figure8D

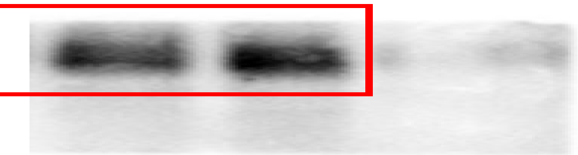

LUAD PDO PD-L1

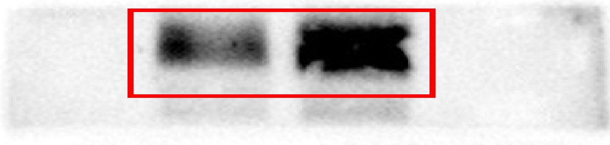

RCC PDO PD-L1

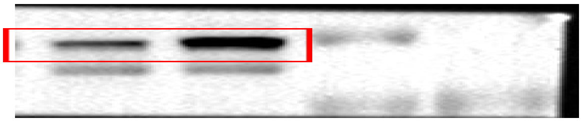

LUAD PDO P-STAT3(Y705)

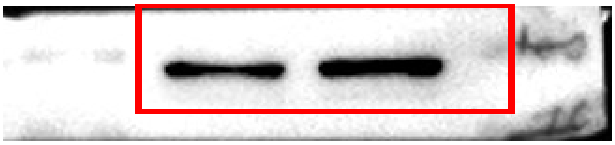

RCC PDO P-STAT3(Y705)

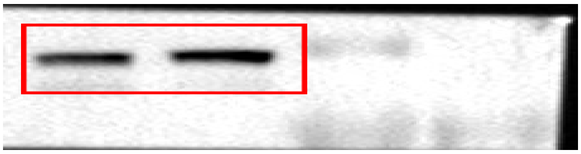

LUAD PDO STAT3

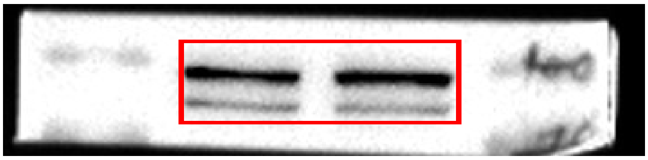

RCC PDO STAT3

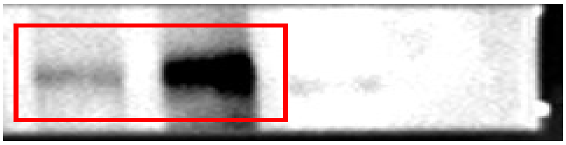

LUAD PDO P-EGFR(Y1068)

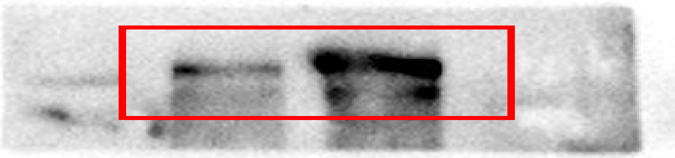

RCC PDO P-EGFR(Y1068)

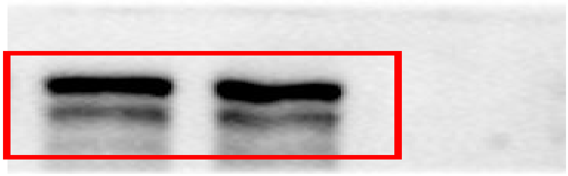

LUAD PDO EGFR

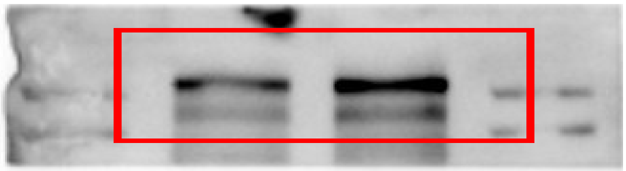

RCC PDO EGFR

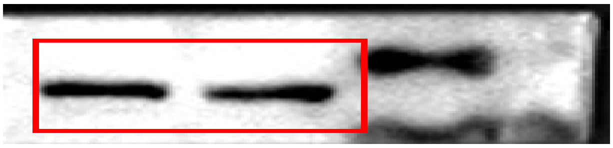

LUAD PDO MFN2

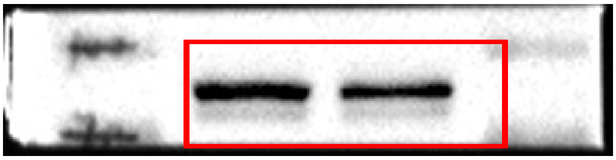

RCC PDO MFN2

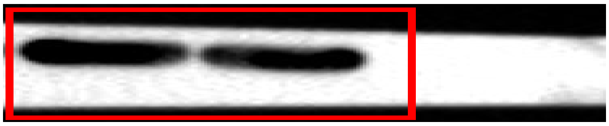

LUAD PDO GAPDH

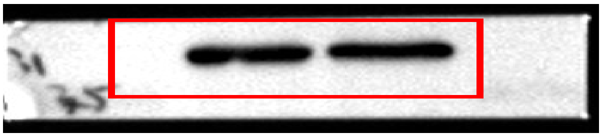

RCC PDO GAPDH

FigureS1B

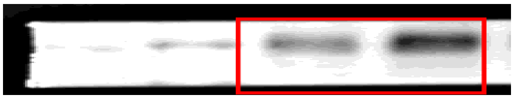

LLC PD-L1

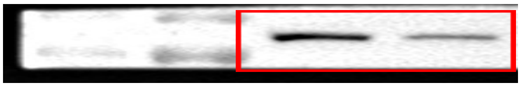

LLC MFN2

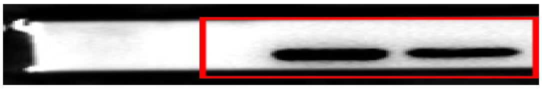

LLC GAPDH

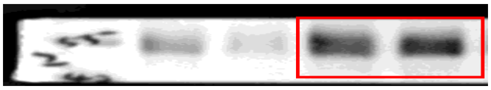

RENCA PD-L1

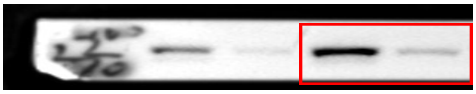

RENCA MFN2

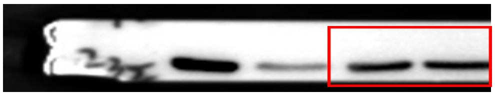

RENCA GAPDH

FigureS2A

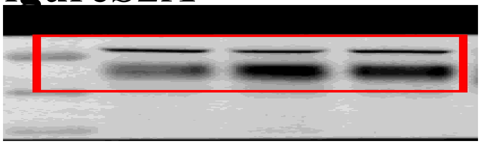

H1299 PD-L1

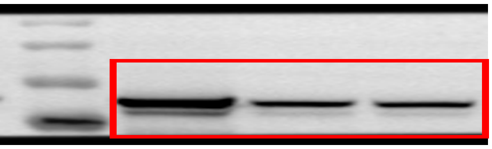

H1299 MFN2

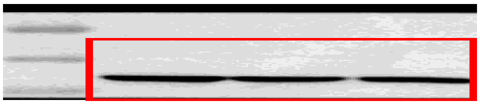

H1299 GAPDH

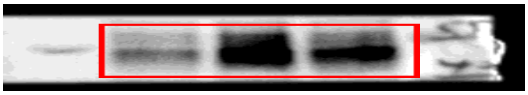

PC-9 PD-L1

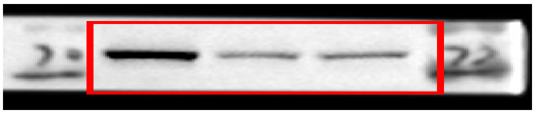

PC-9 MFN2

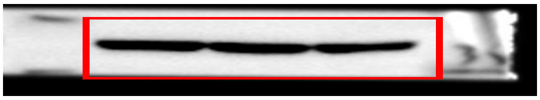

PC-9 GAPDH

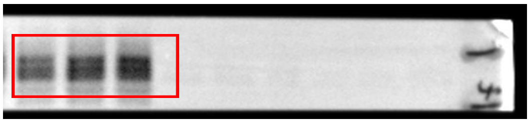

H1975 PD-L1

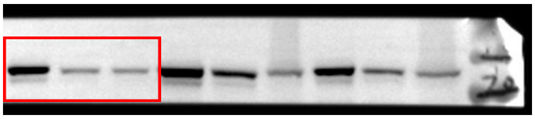

H1975 MFN2

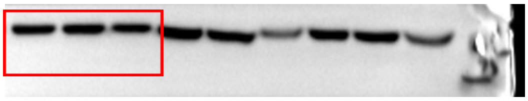

H1975 GAPDH

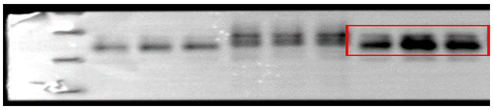

HCC827 PD-L1

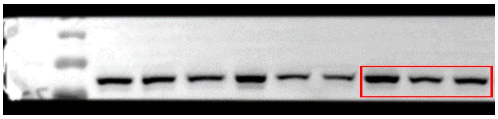

HCC827 MFN2

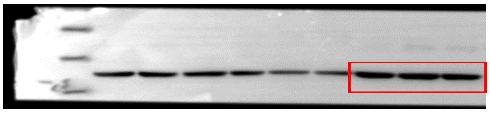

HCC827 GAPDH

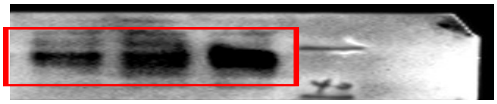

H1703 PD-L1

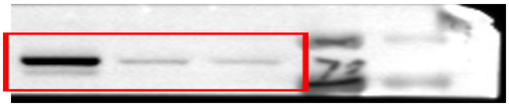

H1703 MFN2

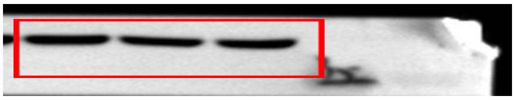

H1703 GAPDH

FigureS2B

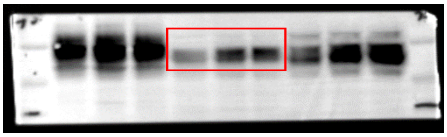

769-P PD-L1

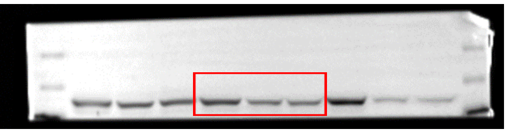

769-P MFN2

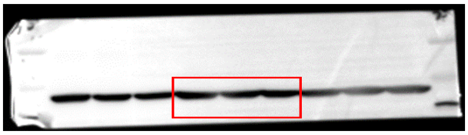

769-P GAPDH

FigureS2C

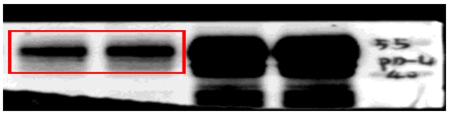

MD-MB-231 PD-L1

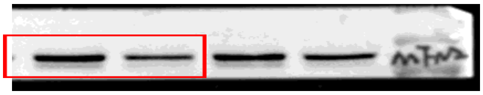

MD-MB-231 MFN2

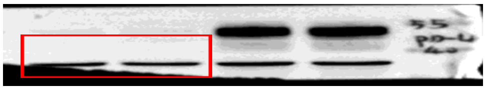

MD-MB-231 GAPDH

FigureS4B

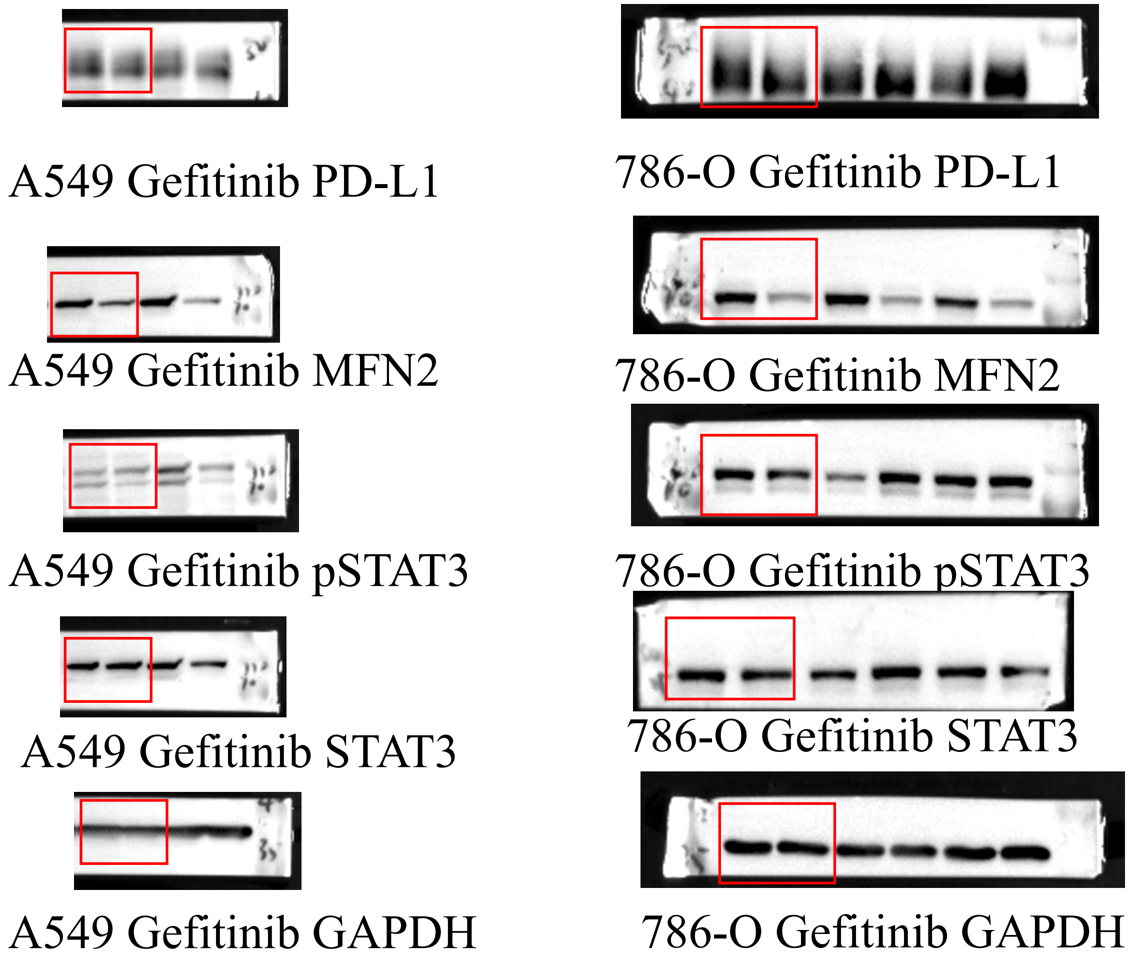
A549 Gefitinib PD-L1
786-O Gefitinib PD-L1
A549 Gefitinib MFN2
786-O Gefitinib MFN2
A549 Gefitinib pSTAT3
786-O Gefitinib pSTAT3
A549 Gefitinib STAT3
786-O Gefitinib STAT3
A549 Gefitinib GAPDH
786-O Gefitinib GAPDH

FigureS4C

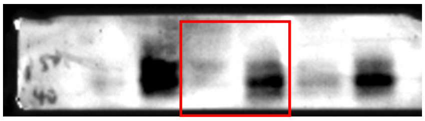

A549 Cabozantinib PD-L1

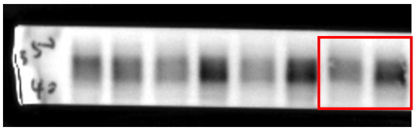

A549 Lenvatinib PD-L1

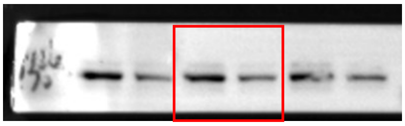

A549 Cabozantinib MFN2

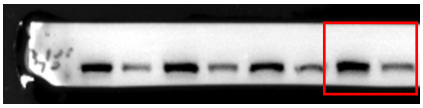

A549 Lenvatinib MFN2

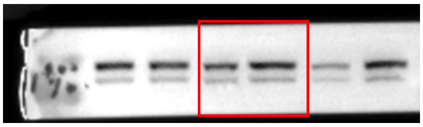

A549 Cabozantinib pSTAT3

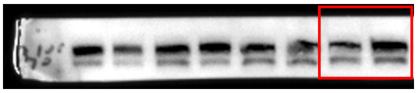

A549 Lenvatinib pSTAT3

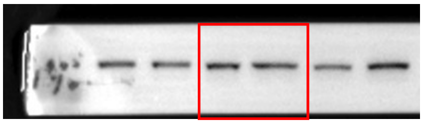

A549 Cabozantinib STAT3

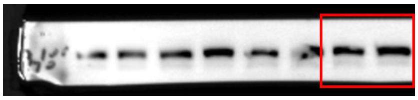

A549 Lenvatinib STAT3

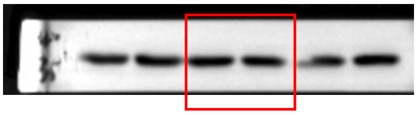

A549 Cabozantinib GAPDH

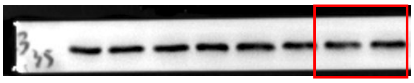

A549 Lenvatinib GAPDH

FigureS4C

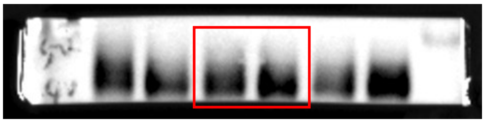

786-O Cabozantinib PD-L1

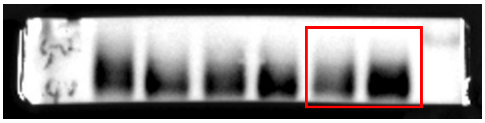

786-O Lenvatinib PD-L1

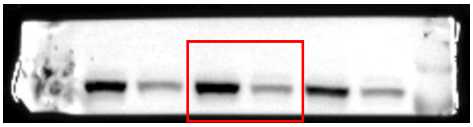

786-O Cabozantinib MFN2

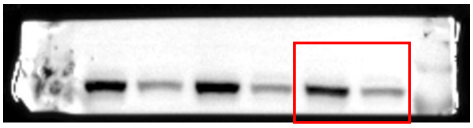

786-O Lenvatinib MFN2

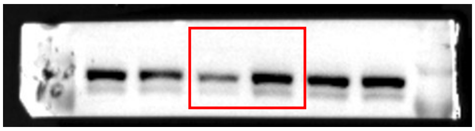

786-O Cabozantinib pSTAT3

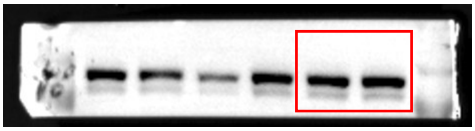

786-O Lenvatinib pSTAT3

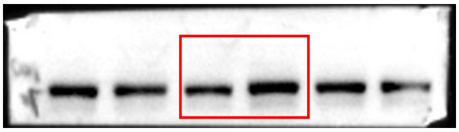

786-O Cabozantinib STAT3

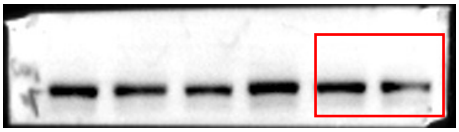

786-O Lenvatinib STAT3

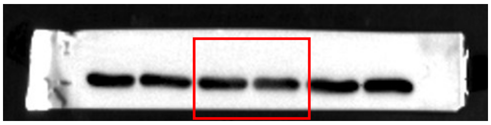

786-O Cabozantinib GAPDH

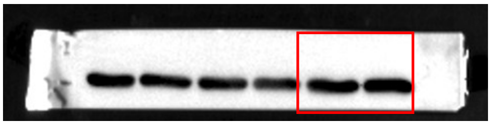

786-O Lenvatinib GAPDH

FigureS4D

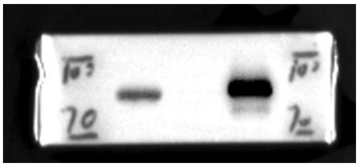

786-O MFN2

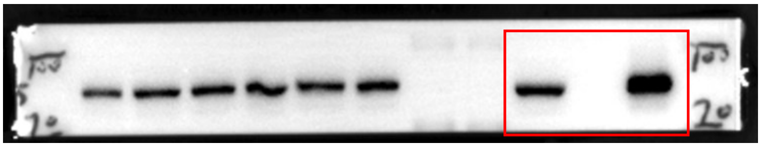

A549 MFN2

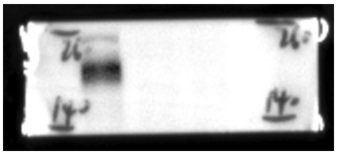

786-O pEGFR

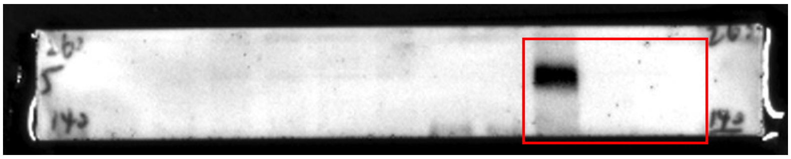

A549 pEGFR

FigureS4E

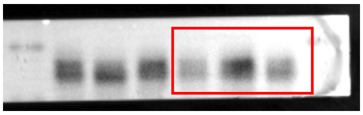

786-O S31-201 PD-L1

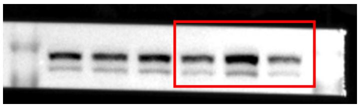

786-O S31-201 pSTAT3

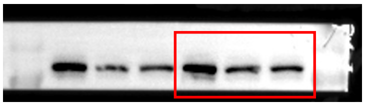

786-O S31-201 MFN2

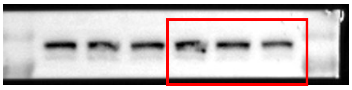

786-O S31-201 STAT3

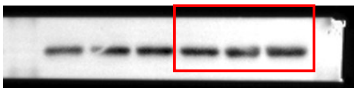

786-O S31-201 GAPDH
