# Supplementary figures and images for: Mitofusin-2 suppresses tumor immune escape through EGFR/STAT3-mediated PD-L1 transcription
Source: Cell Death Dis. 2026 Mar 27;17(1):364. doi: 10.1038/s41419-026-08668-3 (PMC13039860; doi:10.1038/s41419-026-08668-3)

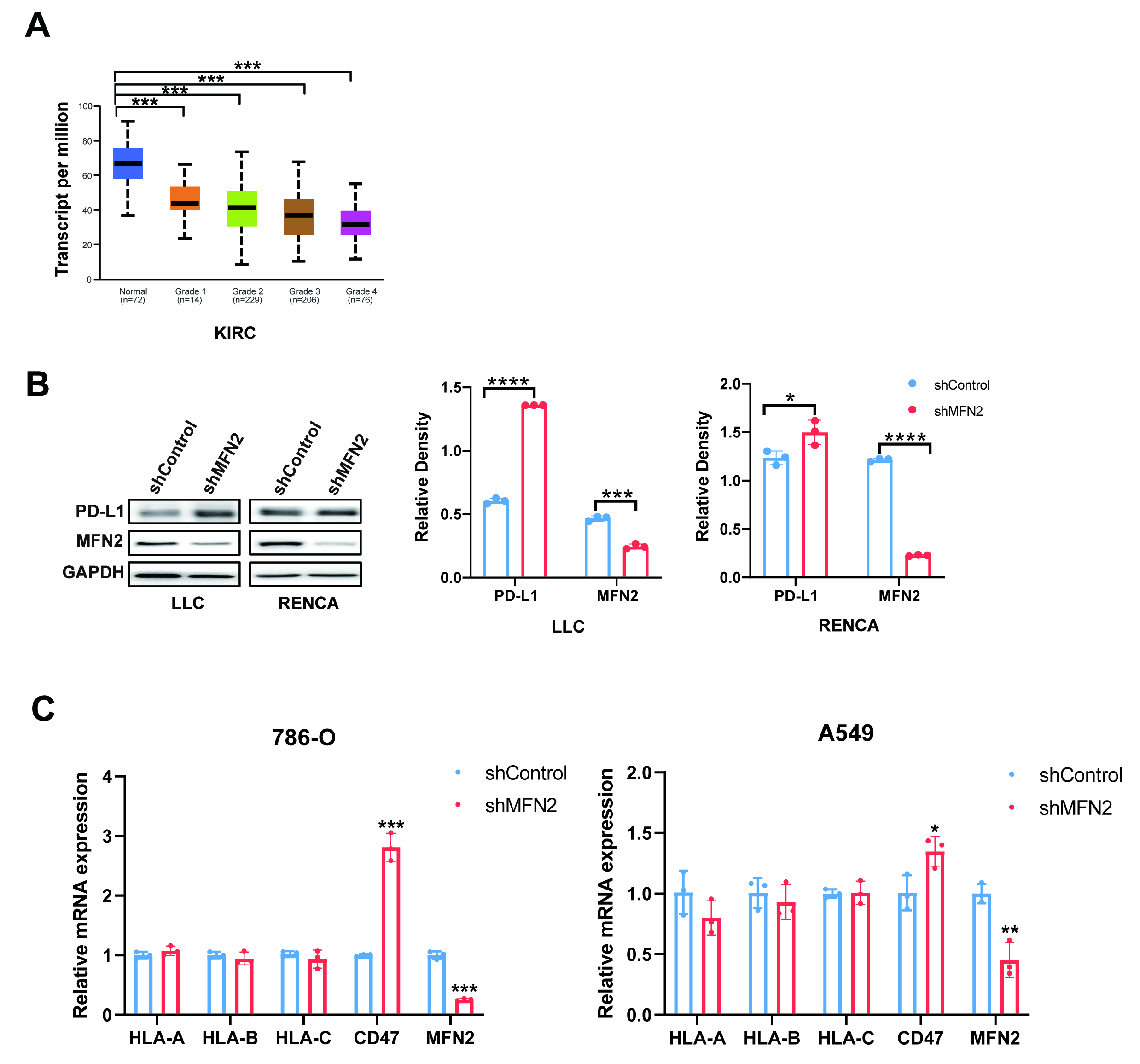

Supplement: Supplementary file 2 — FigureS1 [file 41419_2026_8668_MOESM2_ESM.tif]

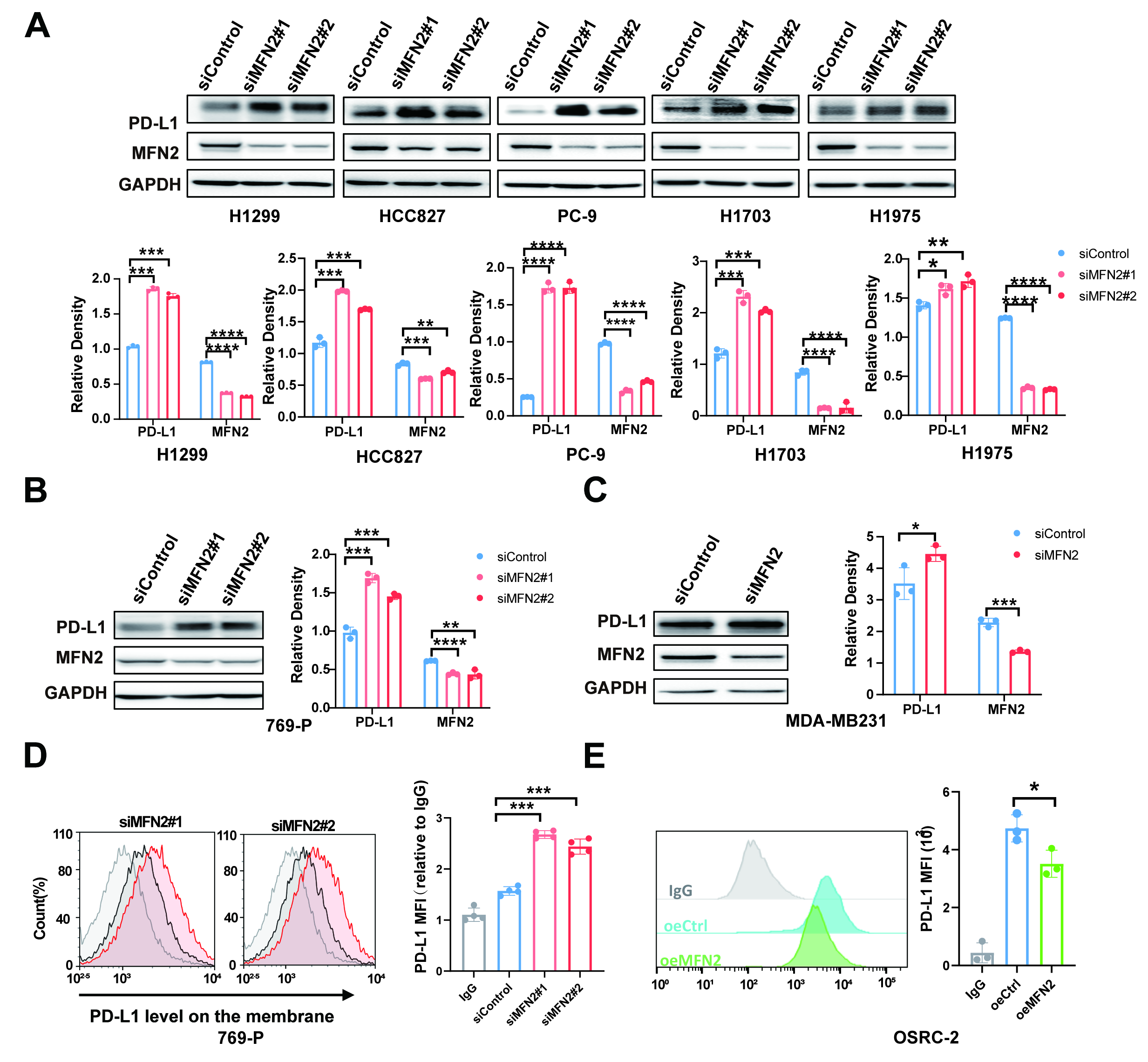

Supplement: Supplementary file 3 — FigureS2 [file 41419_2026_8668_MOESM3_ESM.tif]

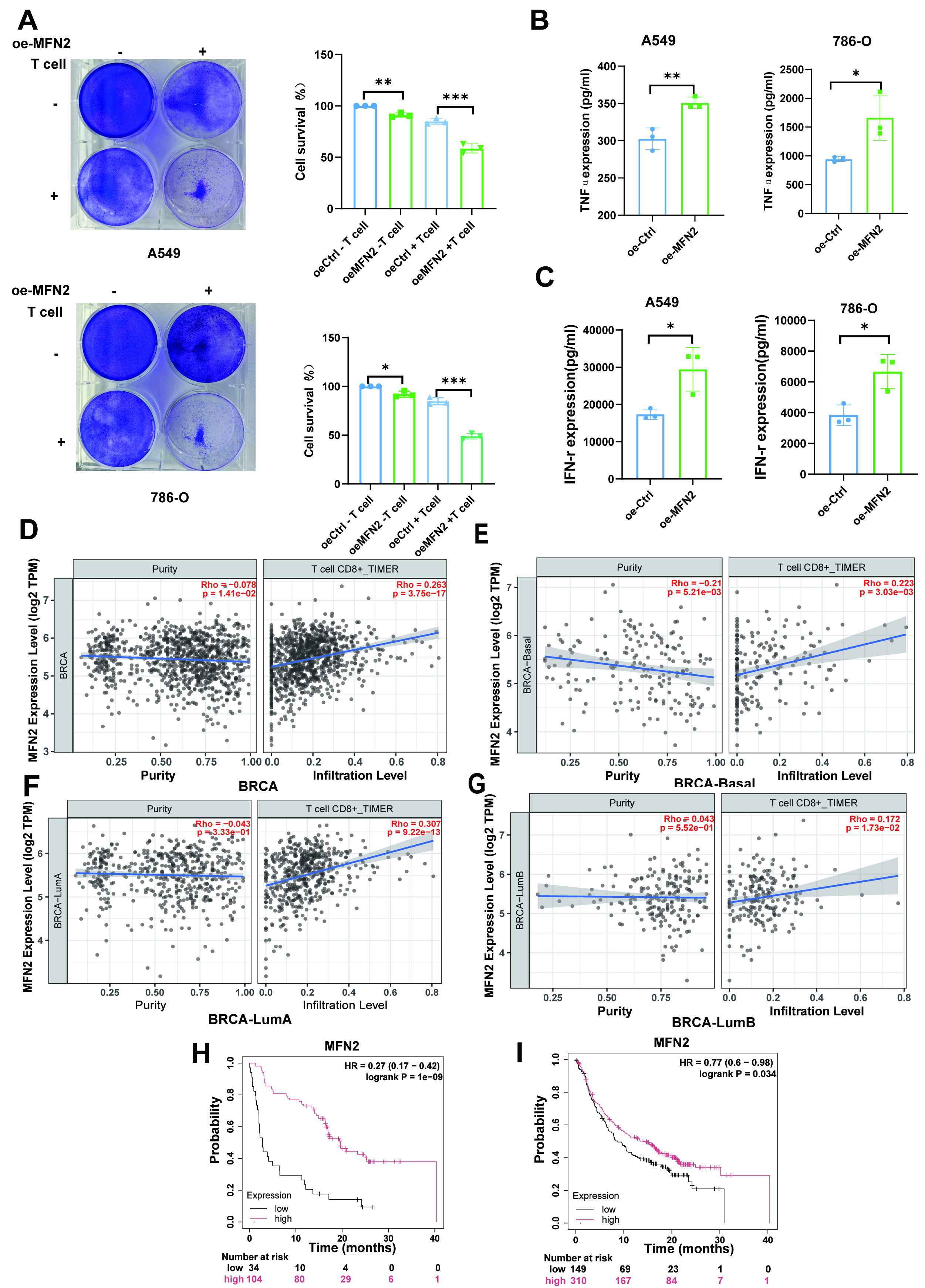

Supplement: Supplementary file 4 — FigureS3 [file 41419_2026_8668_MOESM4_ESM.tif]

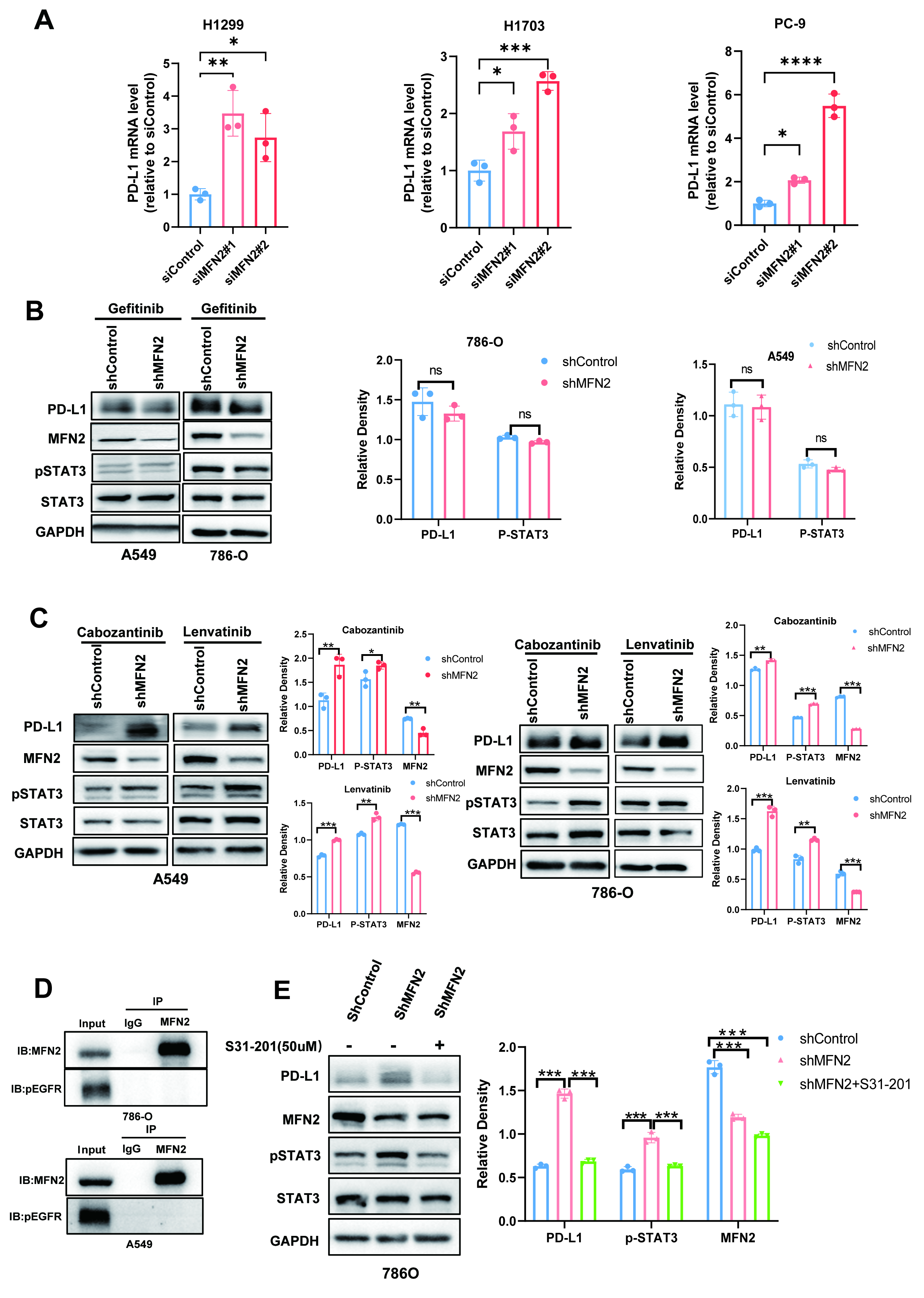

Supplement: Supplementary file 5 — FigureS4 [file 41419_2026_8668_MOESM5_ESM.tif]

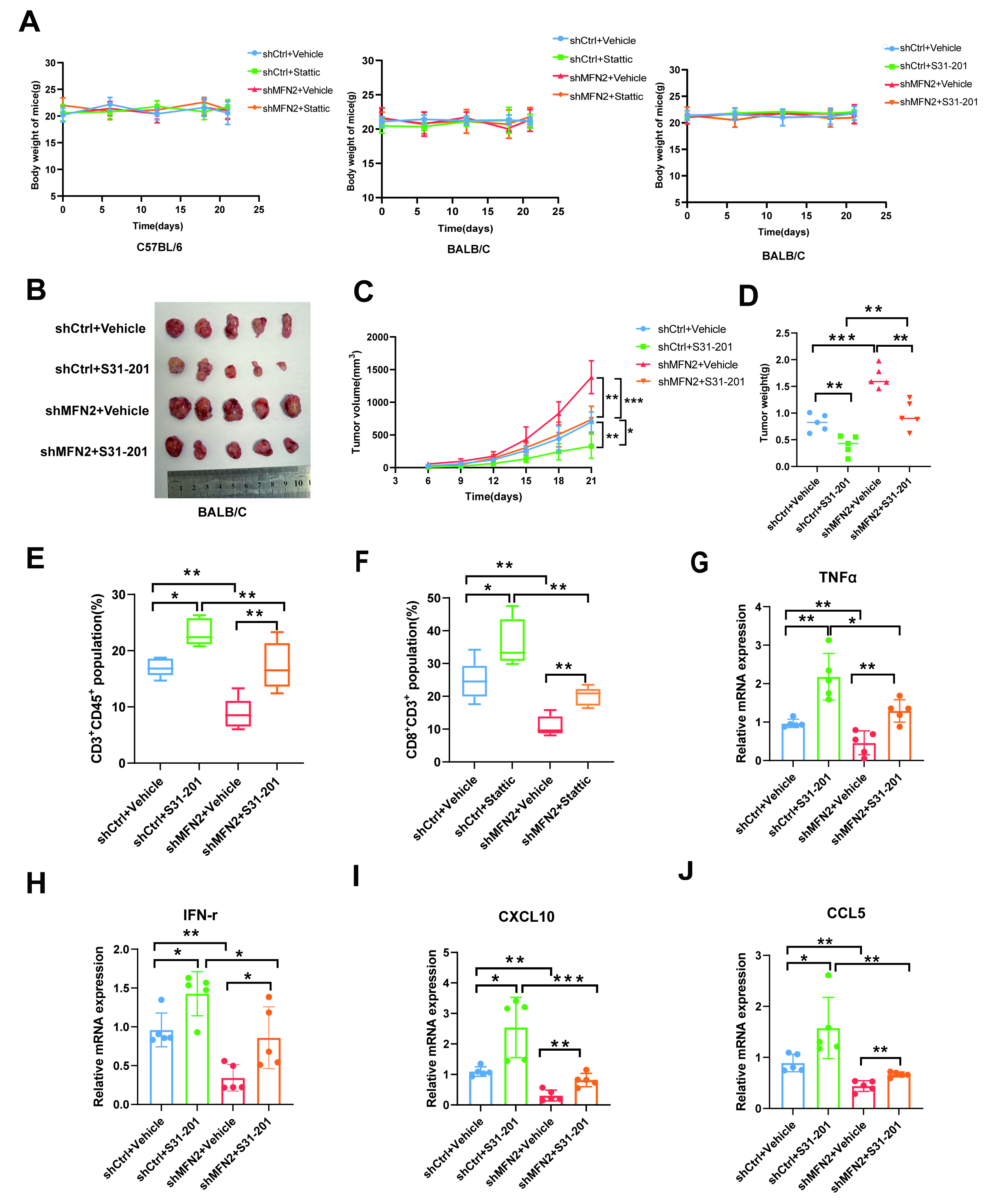

Supplement: Supplementary file 6 — FigureS5 [file 41419_2026_8668_MOESM6_ESM.tif]

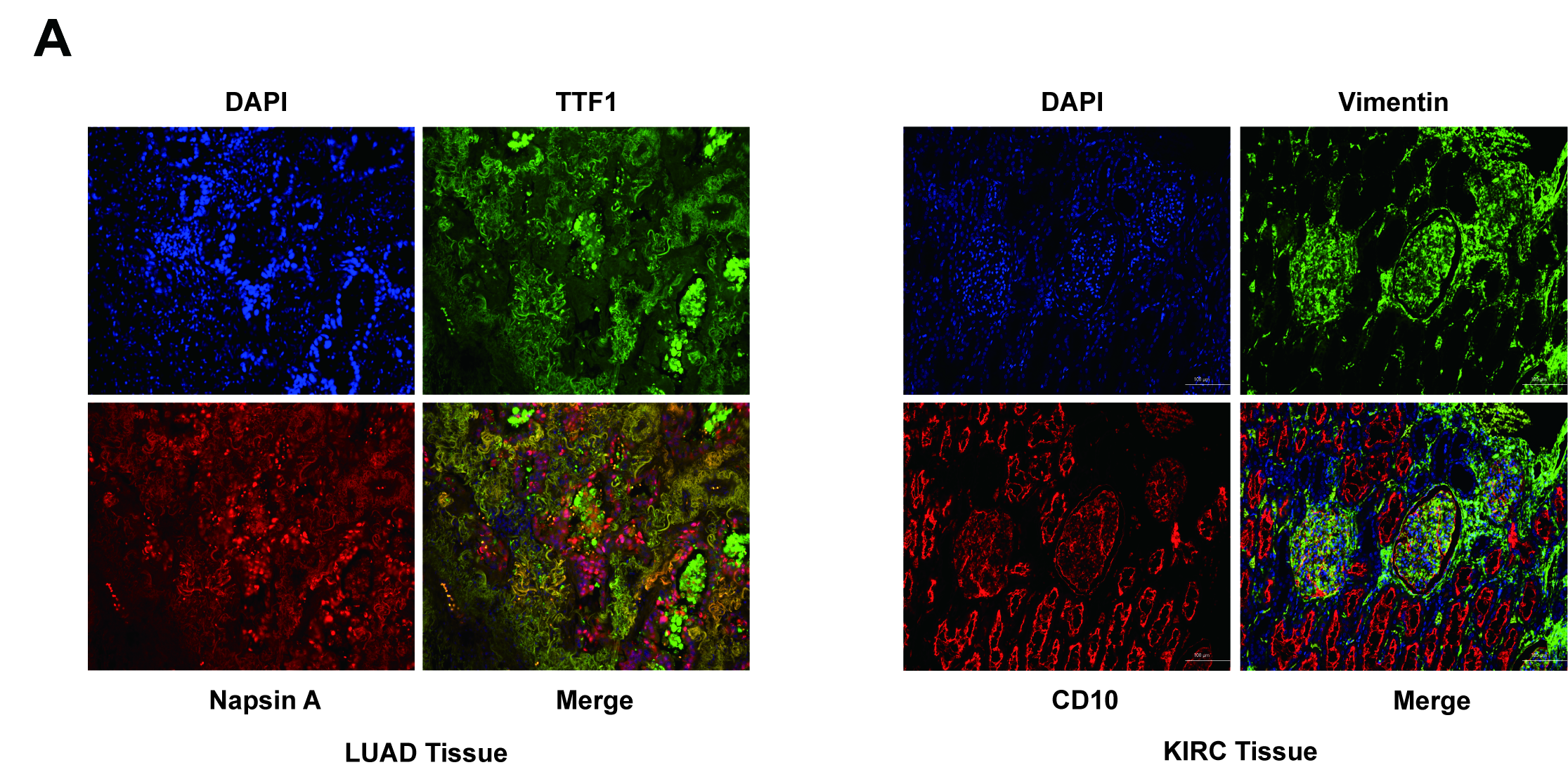

Supplement: Supplementary file 7 — FigureS6 [file 41419_2026_8668_MOESM7_ESM.tif]
