# Supplementary material for: Mitofusin-2 suppresses tumor immune escape through EGFR/STAT3-mediated PD-L1 transcription
Source: Cell Death Dis. 2026 Mar 27;17(1):364. doi: 10.1038/s41419-026-08668-3 (PMC13039860; doi:10.1038/s41419-026-08668-3)
Supplement: Supplementary file 8 — CDDIS-25-6343_Supplementary Figure [file 41419_2026_8668_MOESM8_ESM.docx]

**Figure S1: MFN2 and PD-L1 expression are inversely correlated, with lower MFN2 linked to poorer overall survival in lung and kidney cancer patients.** (A) TCGA analysis showing the association between MFN2 expression and tumor grade based on UALCAN database. (B) Immunoblots showing PD-L1 and MFN2 expression in control and MFN2-knockdown RENCA and LLC cells, Student’s *t* test.(C) RT-PCR analysis showing mRNA expression levels following MFN2 silencing in 786-O and A549 cells, Student’s t test. Data were shown as mean ± SEM; **P* < 0.05, ***P* < 0.01, ****P* < 0.001, *****P* < 0.0001.

**Figure S2: MFN2 regulates PD-L1 expression in multiple cancer cells.** (A)Western blot analysis showing PD-L1 protein levels following MFN2 knockdown in H1299, HCC827, PC-9, H1703 and H1975 cells, One-way ANOVA followed by Tukey’s test. (B) Western blot analysis showing PD-L1 protein levels following MFN2 knockdown in 769-P cells, One-way ANOVA followed by Tukey’s test, Student’s *t* test. (C) Western blot analysis showing PD-L1 protein levels following MFN2 knockdown in MD-MB-231 cells, One-way ANOVA followed by Tukey’s test. (D) Flow cytometric analysis of PD-L1 surface expression in MFN2-knockdown 769-P cells, One-way ANOVA followed by Tukey’s test. (E)Flow cytometric analysis of PD-L1 surface expression in OS-RC-2 cells following MFN2 overexpression, One-way ANOVA followed by Tukey’s test. Data were shown as mean ± SEM; **P* < 0.05, ***P* < 0.01, ****P* < 0.001, *****P* < 0.0001.

**Figure S3:** **Loss of MFN2 Impairs CD8⁺ T-cell Infiltration and Cytotoxic Activity.** (A)Crystal violet staining of oe-control or oe-MFN2 A549 and 786-O cells co-cultured with activated T cells for 48 hours(n=3). The ratio of A549 and 786-O cells to T cells was 1:3. ELISA analysis of TNF-α (B) and IFN-γ (C)expression in the supernatant after co-culture of activated T cells with A549 and 786-O oe-control or oe-MFN2 cells(n=3), Student’s t test. TIMER database analysis showing the correlation between MFN2 expression and CD8^+^ T cell infiltration in BRCA(D), BRCA-Basal(E), BRCA-LumA(F), and BRCA-LumB(G). KM Plot analysis from the KM-Plotter database shows that patients with high expression of MFN2 exhibit improved Progression-Free Survival (PFS) (H) and Overall Survival (OS) (I) following anti-PD-L1 immunotherapy. Kaplan-Meier analysis along with log-rank test. Data were shown as mean ± SEM; **P* < 0.05, ***P* < 0.01, ****P* < 0.001.

**Figure S4: MFN2 regulates PD-L1 expression by activating the EGFR/STAT3 pathway.** (A) RT-PCR analysis showing PD-L1 mRNA expression levels following MFN2 silencing in H1299, H1703, and PC-9 cells, One-way ANOVA followed by Tukey’s test. (B) Western blotting analysis of indicated proteins in MFN2 knockdown A549 cells and 786-O cells treated with Gefitinib (n=3), Student’s *t* test. (C) Western blotting analysis of indicated proteins in MFN2 knockdown A549 cells and 786-O cells treated with Cabozantinib and Lenvatinib (n=3), Student’s *t* test. (D) Co-IP of endogenous p-EGFR with overexpressed MFN2-Flag. Whole cell lysates were prepared from 786-O cells and A549 cells stably expressing MFN2-Flag. Co-IP and Western blotting were performed with the indicated antibodies. (E) Western blot analysis of PD-L1 expression following S31-201 treatment in MFN2-knockdown 786-O cells, One-way ANOVA followed by Tukey’s test(n=3). Data were shown as mean ± SEM; **P* < 0.05, ***P* < 0.01, ****P* < 0.001, *****P* < 0.0001.

**Figure S5. Inhibition of p-STAT3 suppresses MFN2 knockdown–induced tumor growth and immune evasion.** (A)Mouse body weights were measured regularly throughout the treatment period, Two-way ANOVA. (B) Tumor mass of dissected tumors from mice across different intervention groups. (C) Tumor growth curves showing mean tumor volume over time in mice bearing subcutaneous tumors under different treatment regimens, Two-way ANOVA(n=5). (D) Comparison of tumor weights among different intervention groups in mice, One-way ANOVA followed by Tukey’s test(n=5). The RT-qPCR analysis of the expressions of IFN-γ (E), TNF-α (F), CCL-5 (G), and CXCL-10 (H) in bulk RENCA tumor xenografts, One-way ANOVA followed by Tukey’s test(n=5). (I) Proportion of CD3^+^CD45^+^T lymphocytes in tumor tissues from different treatment groups, One-way ANOVA followed by Tukey’s test(n=5). (J) Proportion of CD8^+^CD3^+^T lymphocytes in tumor tissues from different treatment groups, One-way ANOVA followed by Tukey’s test. Data were shown as mean ± SEM; **P* < 0.05, ***P* < 0.01, ****P* < 0.001

**Figure S6. Validation of MFN2-mediated regulation of PD-L1 expression via the EGFR/STAT3 signaling pathway at the tumor organoid level.** (A) Immunofluorescence confocal identification of organoid models derived from human KIRC and LUAD tissues.
