# Supplementary material for: Mitofusin-2 suppresses tumor immune escape through EGFR/STAT3-mediated PD-L1 transcription
Source: Cell Death Dis. 2026 Mar 27;17(1):364. doi: 10.1038/s41419-026-08668-3 (PMC13039860; doi:10.1038/s41419-026-08668-3)
Supplement: Supplementary file 9 — CDDIS-25-6343_Supplementary Table [file 41419_2026_8668_MOESM9_ESM.docx]

**Supplementary Table 1. Antibody Information**

| Antibody | Source | Identifier | Dilutions |
| --- | --- | --- | --- |
| anti-MFN2(human) | CST | Cat#O95140 | 1:1000 for WB |
| anti-PD-L1(human) | Abcam | Cat#Ab237726 | 1:1000 for WB |
| anti-EGFR | CST | Cat #2232 | 1:1000 for WB |
| anti-p-EGFR | CST | Cat#P00533 | 1:1000 for WB |
| anti-Stat3 | CST | Cat #9132 | 1:1000 for WB |
| anti-p-Stat3 | CST | Cat#P40763 | 1:2000 for WB |
| anti-Tublin | Proteintech | Cat#11224-1-AP | 1:10000 for WB |
| anti-PD-L1(mouse) | Abcam | Cat#ab210931 | 1:1000 for WB |
| anti-GAPDH | Proteintech | Cat#60004-1-Ig | 1:20000 for WB |
| anti-Histone H3 | CST | Cat#P68431 | 1:1000 for WB |
| anti-CD10 | Biolynx | Cat#bx50054-c3 | 1:200 for IF |
| anti-TTF1 | Biolynx | Cat#bx50074 | 1:200 for IF |
| anti-Napsin A | Biolynx | Cat# bx50078 | 1:200 for IF |
| anti-Vimentin | Biolynx | Cat# bx50006-c3 | 1:200 for IF |
| anti-CD45-PerCP | eBioscience | Cat# F1136J | 1:20 for FC |
| anti-CD3-PE | eBioscience | Cat# F1013D | 1:20 for FC |
| anti-CD4-FITC | eBioscience | Cat# F1353C | 1:20 for FC |
| anti-CD8a-APC | eBioscience | Cat# F1104E | 1:20 for FC |
| PE anti-human CD274 | Biolegend | Cat# 374511 | 1:20 for FC |
| goat anti-rabbit IgG H&L(Alexa Fluor 488) | abcam | Cat#ab150077 | 1:400 for IF |
| goat anti-rabbit IgG H&L(Alexa Fluor 594) | abcam | Cat#ab150080 | 1:400 for IF |
| goat anti-mouse IgG H&L(Alexa Fluor 488) | abcam | Cat#ab150113 | 1:400 for IF |
| goat anti-mouse IgG H&L(Alexa Fluor 594) | abcam | Cat#ab150116 | 1:400 for IF |

**Supplementary Table 2. Sequences of Oligonucleotides for qPCR and RNA Interference.**

| Oligonucleotides Resource | Sequence | Application |
| --- | --- | --- |
| si-hMFN2#1-F | 5’-GCAGGUUUACUGCGAGGAAAUTT-3’ | RNA interference |
| si-hMFN2#1-R | 5’-AUUUCCUCGCAGUAAACCUGCTT-3’ | RNA interference |
| si-hMFN2#2-F | 5’-GUCAAAGGUUACCUAUCCAAATT-3’ | RNA interference |
| si-hMFN2#2-R | 5’-UUUGGAUAGGUAACCUUUGACTT-3’ | RNA interference |
| si-hStat3-F | 5′-CCCGGAAATTTA ACATTCT-3′ | RNA interference |
| si-hStat3-R | 5′-AGAAUGUUAAAUUUCCGGG-3′ | RNA interference |
| si-Control-F | 5‘-UUCUCCGAACGUGUCACGUTT-3′ | RNA interference |
| si-Control-R | 5‘-ACGUGACACGUUCGGAGAATT-3′ | RNA interference |
| Sh-hMFN2-F | 5’-CCGGGTCAAAGGTTACCTATCCAAACTCGAGTTTGGATAGGTAACCTTTGACTTTTTG-3’ | RNA interference |
| Sh-hMFN2-R | 5’-CAGTTTCCAATGGATAGGTTTGAGCTCAAACCTATCCATTGGAAACTGAAAAACTAA-3’ | RNA interference |
| Sh-hScramble-F | 5’-CCGGCCTAAGGTTAAGTCGCCCTCGCTCGAGCGAGGGCGACTTAACCTTAGGTTTTTG-3’ | RNA interference |
| Sh-hScramble-R | 5’-GGATTCCAATTCAGCGGGAGCGAGCTCCGAGGGCGACTTAACCTTAGGAAAAACTAA-3’ | RNA interference |
| Sh-mMFN2-F | 5’-CCGGGGAAGAGCACCGTGATCAATGCTCGAGCATTGATCACGGTGCTCTTCCTTTTTG-3’ | RNA interference |
| Sh-mMFN2-R | 5’-CCTTCTCGTGGCACTAGTTACGAGCTCGTAACTAGTGCCACGAGAAGGAAAAACTAA-3’ | RNA interference |
| hPD-L1-F | 5’-GCTGCACTAATTGTCTATTGGGA-3’ | RT-qPCR primer |
| hPD-L1-R | 5’-AATTCGCTTGTAGTCGGCACC-3’ | RT-qPCR primer |
| hMFN2-F | 5’-CTCTCGATGCAACTCTATCGTC-3’ | RT-qPCR primer |
| hMFN2-R | 5’-TCCTGTACGTGTCTTCAAGGAA-3’ | RT-qPCR primer |
| mIFN-r-F | 5’-ACAGCAAGGCGAAAAAGGATG-3’ | RT-qPCR primer |
| mIFN-r-R | 5’-TGGTGGACCACTCGGATGA-3’ | RT-qPCR primer |
| mTNF-a-F | 5’-CCCTCACACTCAGATCATCTTCT-3’ | RT-qPCR primer |
| mTNF-a-R | 5’-GCTACGACGTGGGCTACAG-3’ | RT-qPCR primer |
| mCCL-5-F | 5’-GCTGCTTTGCCTACCTCTCC-3’ | RT-qPCR primer |
| mCCL-5-R | 5’-TCGAGTGACAAACACGACTGC-3’ | RT-qPCR primer |
| mCXCL-10-F | 5’-TGAATCCGGAATCTAAGACCATCAA-3’ | RT-qPCR primer |
| mCXCL-10-R | 5’-AGGACTAGCCATCCACTGGGTAAAG-3’ | RT-qPCR primer |
| mβactin-F | 5’-GGCTGTATTCCCCTCCATCG-3’ | RT-qPCR primer |
| mβactin-R | 5’-CCAGTTGGTAACAATGCCATGT-3’ | RT-qPCR primer |
| Homo PD-L1-F | 5’-CAAGGTGCGTTCAGATGTTG-3’ | Primers for ChIP assay |
| Homo PD-L1-R | 5’-GGCGTTGGACTTTCCTGA-3’ | Primers for ChIP assay |

**Supplementary Table 3. The correlation between clinicopathological parametersand MFN2/PD-L1/CD8 expression in LUAD cancer.**

| **MFN2**  **Low n(%) high n(%) *P*** | | | |  | **PD-L1**  **Low n(%) high n(%) *P*** | | | |  | **CD8**  **Low n(%) high n(%) *P*** | | | |
| --- | --- | --- | --- | --- | --- | --- | --- | --- | --- | --- | --- | --- | --- |
| **Age** |  | | |  | Age |  | | |  | Age |  | | |
| **>50(years)** | 15（30%） | 13（26%） | p=0.776 |  | >50(years) | 14(28%) | 8(16%) | p=0.1536 |  | >50(years) | 13(26%) | 11(22%) | p=0.775 |
| **≤50(years)** | 10（20%） | 12（24%) |  |  | ≤50(years) | 11(22%) | 17(34%) |  |  | ≤50(years) | 12(24%) | 14(28%) |  |
| **Gender** |  | | |  | Gender |  | | |  | Gender |  | | |
| **Male** | 15（30%） | 11(22%) | p=0.3961 |  | Male | 19(38%) | 18(36%) | p=0.7735 |  | Male | 17(34%) | 16(32%) | >0.9999 |
| **female** | 10（20%） | 14(28%) |  |  | female | 6(12%) | 7(14%) |  |  | female | 8(16%) | 9(18%) |  |
| **T stage** |  | | |  | T stage |  | | |  | T stage |  | | |
| **T1-T2** | 22(44%) | 21(42%) | >0.9999 |  | T1-T2 | 21(42%) | 20(40%) | >0.9999 |  | T1-T2 | 22(44%) | 21(42%) | >0.9999 |
| **T3-T4** | 3(6%) | 4(8%) |  |  | T3-T4 | 4(8%) | 5(10%） |  |  | T3-T4 | 3(6%) | 4(8%) |  |
| **N stage** |  | | |  | N stage |  | | |  | N stage |  | | |
| **N0** | 15（30%） | 17(34%) | 0.7688 |  | N0 | 21(42%) | 15（30%） | 0.1137 |  | N0 | 14(28%) | 16(32%) | 0.7733 |
| **N1-NX** | 10（20%） | 8(16%) |  |  | N1-NX | 4(8%) | 10（20%） |  |  | N1-NX | 11(22%) | 9(18%) |  |

**Supplementary Table 4. The correlation between clinicopathological parametersand MFN2/PD-L1/CD8 expression in KIRC cancer.**

| **MFN2**  **Low n(%) high n(%) *P*** | | | |  | **PD-L1**  **Low n(%) high n(%) *P*** | | | |  | **CD8**  **Low n(%) high n(%) *P*** | | | |
| --- | --- | --- | --- | --- | --- | --- | --- | --- | --- | --- | --- | --- | --- |
| **Age** |  | | |  | Age |  | | |  | Age |  | | |
| **>50(years)** | 15（30%） | 13（26%） | 0.7761 |  | >50(years) | 18(36%) | 14(28%) | 0.3772 |  | >50(years) | 17(34%) | 12（24%) | 0.2516 |
| **≤50(years)** | 10（20%） | 12（24%) |  |  | ≤50(years) | 7(14%) | 11(22%) |  |  | ≤50(years) | 8(16%) | 13（26%） |  |
| **Gender** |  | | |  | Gender |  | | |  | Gender |  | | |
| **Male** | 11(22%) | 9(18%) | 0.7733 |  | Male | 11(22%) | 7(14%) | 0.3772 |  | Male | 10（20%） | 7(14%) | 0.5512 |
| **female** | 14(28%) | 16(32%) |  |  | female | 14(28%) | 18(36%) |  |  | female | 15（30%） | 18(36%) |  |
| **T stage** |  | | |  | T stage |  | | |  | T stage |  | | |
| **T1-T2** | 20(40%) | 13(26%) | 0.0718 |  | T1-T2 | 22(44%) | 17(34%) | 0.1706 |  | T1-T2 | 19(38%) | 18(36%) | >0.9999 |
| **T3-T4** | 5(10%） | 12(24%) |  |  | T3-T4 | 3(6%) | 8(16%) |  |  | T3-T4 | 6(12%) | 7(14%) |  |
| **N stage** |  | | |  | N stage |  | | |  | N stage |  | | |
| **N0** | 2（4%） | 3(6%) | >0.9999 |  | N0 | 4(8%) | 3(6%) | >0.9999 |  | N0 | 2（4%） | 4(8%) | 0.6671 |
| **N1-NX** | 23（46%） | 22(44%) |  |  | N1-NX | 21(42%) | 22(44%) |  |  | N1-NX | 23（46%） | 21(42%) |  |
